# Matrix stiffness regulates glucose-6-phosphate dehydrogenase expression to mediate sorafenib resistance in hepatocellular carcinoma through the ITGB1-PI3K/AKT pathway

**DOI:** 10.1038/s41419-025-07842-3

**Published:** 2025-07-20

**Authors:** Ruimei Ren, Shan Zhang, Zhan Peng, Xiaomeng Ji, Hao Song, Qiuxiao Wang, Xiangyin Sun, Huiyu Wang, Yinying Dong

**Affiliations:** 1 Department of Radiation Oncology, The Affiliated Hospital of Qingdao University, Qingdao, China; 2 State Key Laboratory of Systems Medicine for Cancer, Shanghai Cancer Institute, Ren Ji Hospital, School of Medicine, Shanghai Jiao Tong University, Shanghai, P.R. China; 3 Department of Oncology, Qingdao Sixth People’s Hospital, Qingdao, China; 4 Department of Radiation Oncology, Qingdao Chengyang District People’s Hospital, Qingdao, China

**Keywords:** Cancer microenvironment, Cell growth

## Abstract

Sorafenib is an antiangiogenic and antiproliferative chemotherapeutic drug that plays a crucial role in the treatment of patients with advanced hepatocellular carcinoma (HCC). However, resistance to sorafenib greatly limits its therapeutic efficacy. This highlights the importance of determining the mechanisms underlying resistance to antiangiogenic therapy. In this study, we found that the extracellular matrix (ECM) stiffness was closely related to the prognosis of HCC patients and chemotherapy resistance. Using atomic force microscopy, we assessed ECM stiffness in tumor samples from 30 HCC patients treated with sorafenib, and the ECM stiffness in sorafenib-resistant patients was significantly greater than that in those who responded to sorafenib treatment. In a liver orthotopic xenograft model, reducing tumor ECM stiffness by inhibiting LOX enzyme activity significantly enhanced the efficacy of sorafenib and suppressed tumor progression. We found that glucose-6-phosphate dehydrogenase (G6PD) is regulated by ECM stiffness and is involved in resistance to sorafenib. Further in vitro and in vivo experiments confirmed that ECM stiffness can upregulate G6PD expression through the ITGB1-PI3K/AKT pathway, mediating sorafenib resistance in HCC. Clinical tissue microarray analysis revealed that the expression of collagen I, α-SMA, ITGB1, p-AKT, and G6PD was associated with sorafenib resistance in HCC patients. These results indicated that reducing ECM stiffness can increase the sensitivity of HCC to sorafenib and that the ITGB1-PI3K/AKT-G6PD cascades may serve as potential therapeutic targets for reversing sorafenib resistance.

## Introduction

Hepatocellular carcinoma (HCC) is the predominant subtype of primary liver cancer and ranks as the second leading cause of cancer-related mortality worldwide [1]. The incidence of HCC is increasing globally, and it is projected to surpass one million cases by 2025 [2]. The predominant risk factors for HCC are infections with hepatitis B and C viruses and nonalcoholic steatohepatitis associated with metabolic syndrome or diabetes. In the clinic, more than 90% of HCC cases are accompanied by liver fibrosis and cirrhosis, particularly in cases related to inflammation [3]. In contrast to pancreatic cancer or cholangiocarcinoma, where fibrosis within the tumor environment (TME) occurs due to tumor development, fibrosis precedes the development of HCC [4]. The stiffness of the extracellular matrix (ECM), a critical biomechanical cue in the TME, affects the regulation of multiple biological processes. Dysregulated ECM stiffness plays a role in the initiation and development of HCC by regulating lipid metabolism, angiogenesis, invasion, and metabolic processes [5]. Thus, a better understanding of the mechanisms driving the progression of HCC due to ECM stiffness may improve the clinical outcomes of HCC patients.

Sorafenib is a multitarget tyrosine kinase inhibitor with antiangiogenic and antiproliferative effects. Sorafenib is the first-line treatment for advanced HCC patients and significantly prolongs their total median survival [6]. However, sorafenib resistance is commonly observed in HCC patients. Various biological processes, such as apoptosis, hypoxia, and epigenetic alterations, are involved in sorafenib resistance [7]. Although the mechanisms underlying sorafenib resistance have been investigated in several aspects, the relationship between sorafenib resistance and ECM stiffness remains unknown. Glucose-6-phosphate dehydrogenase (G6PD), the rate-limiting enzyme in the pentose phosphate pathway, is an important source of cellular NADPH and plays an important role in protecting cells from oxidative stress [8]. G6PD is involved in numerous diseases, including viral infections, vascular diseases, seizures, and cancer [9]. G6PD expression is upregulated in several types of cancer and is related to drug resistance [10]. G6PD inhibition can enhance the response to chemotherapy in pancreatic cancer [11]. Additionally, G6PD plays a role in resistance to dexamethasone-induced apoptosis by increasing antioxidant production and activating the Wnt/β-catenin pathway in multiple myeloma [12]. These findings suggest that G6PD may strongly influence HCC progression and sorafenib resistance.

In this study, we found the specific contribution of ECM stiffness to sorafenib resistance in HCC. We also found that G6PD, a key regulator of sorafenib resistance, acts as a downstream molecule of ECM stiffness. Targeting ECM stiffness and G6PD can significantly inhibit cell proliferation and increase the potential of sorafenib therapy in HCC via loss- and gain-of-function studies. We found that ECM stiffness upregulated G6PD expression by activating the ITGB1-PI3K/AKT pathway. Thus, the ECM stiffness-ITGB1-PI3K/AKT-G6PD axis plays a role in sorafenib resistance in HCC.

## Results

### ECM stiffness is related to chemotherapy resistance in HCC

**Fig. 1:**
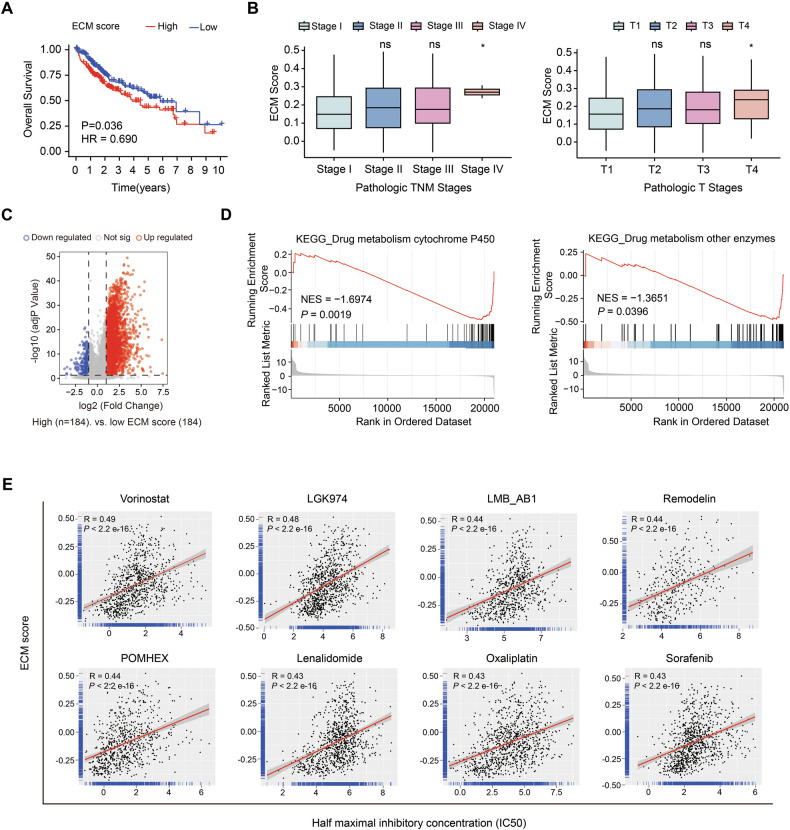
Relationships between the ECM score and clinicopathological parameters in HCC patients. **A** The effect of the ECM score on the overall survival of patients in the TCGA-HCC patient cohort. **B** Association between the ECM score and pathological stage, based on the TCGA database. **C** A volcano plot depicting the DEGs linked to the ECM based on transcriptomic data from the TCGA database. **D** Gene set enrichment analysis (GSEA) plot of drug metabolism cytochrome P450 and other drug metabolism enzyme pathways between the low- and high-ECM stiffness groups. **E** Scatter plot emphasizing the chemotherapeutic drugs most strongly correlated with the ECM score (*R* > 0.43, *P* < 0.05). The ECM score was positively associated with resistance to various chemotherapeutic agents, including vorinostat (HDAC inhibitor), LGK974 (Wnt pathway inhibitor), LMB_AB1 (ADRA1A and ADRB1 inhibitor), Remodelin (N-acetyltransferase 10 inhibitor), POMHEX (phosphonate inhibitor), lenalidomide (targeted immunomodulatory agent), oxaliplatin (DNA synthesis inhibitor), and sorafenib (multitargeted tyrosine kinase inhibitor). ns not significant, *P < 0.05.

To delineate the pathological implications of ECM stiffeness in HCC progression, we performed transcriptomic stratification of 369 HCC specimens from The Cancer Genome Atlas (TCGA) database. Unsupervised hierarchical clustering analysis (Euclidean distance metric, complete linkage method) partitioned samples into mechano-phenotypic subgroups based on expression profiles of ECM stiffness-associated genes. Subsequent consensus clustering refined the classification, yielding two distinct cohorts: high-ECM stiffness (*n* = 183) and low-ECM stiffness (*n* = 184) groups. Survival analysis revealed significant prognostic stratification, with high ECM stiffness correlating with reduced overall survival (OS) [HR = 0.69 (95% CI: 0.489–0.975), *P* = 0.036] (Fig. 1A). Clinicopathological correlation analysis demonstrated matrix stiffeness positively associated with advanced T stage and tumor–node–metastasis (TNM) stage progression (Fig. 1B), suggesting biomechanical remodeling parallels disease aggressiveness. Differential expression analysis (|log_2_FC| > 1, *P* value <0.05) identified 3972 ECM stiffness-associated genes, comprising 3656 upregulated and 316 downregulated transcripts (Fig. 1C). Gene set enrichment analysis (GSEA) of Hallmark pathways revealed strong association between ECM stiffening signatures and chemoresistance mechanisms (Fig. 1D). Validation using the Genomics of Drug Sensitivity in Cancer (GDSC) resource demonstrated significant inverse correlations between ECM stiffness scores and sensitivity to first-line HCC therapeutics, including Oxaliplatin (*R* = 0.43, *P* < 2.2  e^−16^) and Sorafenib (*R* = 0.43, *P* < 2.2  e^−16^) (Fig. 1E and Supplementary Fig. 1). These findings suggested that ECM stiffness is closely related to the aggressive behavior of HCC, particularly in terms of drug resistance.

### ECM stiffness promotes resistance to sorafenib in vivo and in vitro

**Fig. 2:**
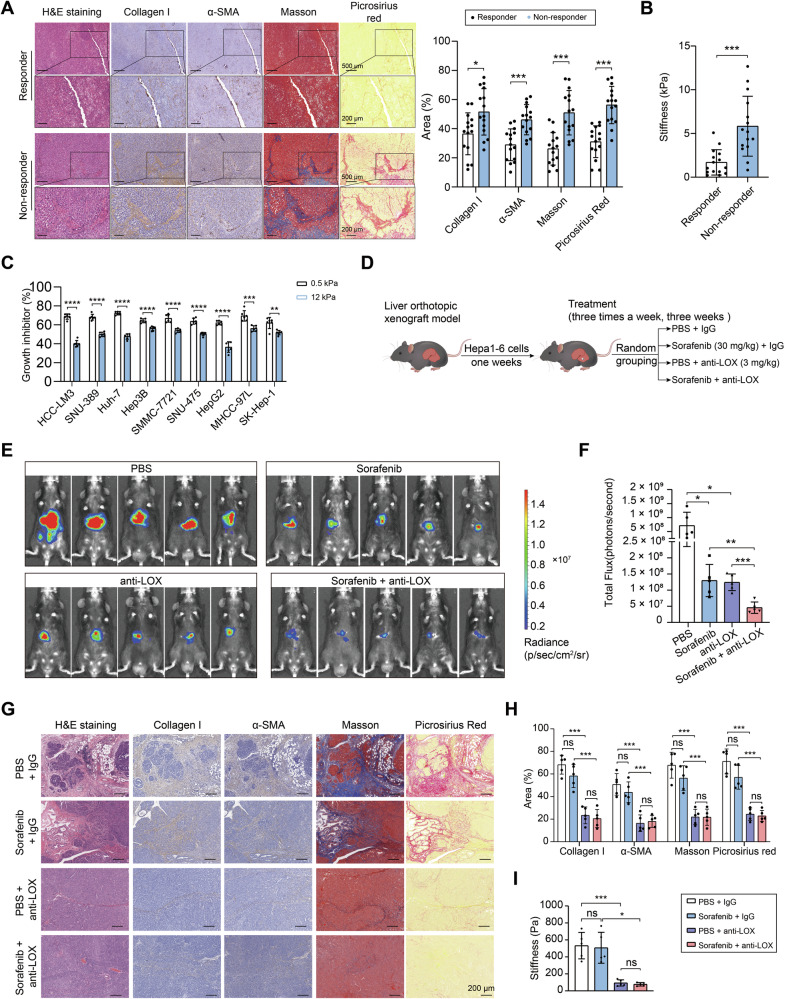
ECM stiffness promotes resistance to sorafenib. **A** Representative images of hematoxylin and eosin (H&E), collagen I, α-SMA, Masson’s trichrome, and Picrosirius red staining of consecutive pathological sections showing ECM deposition in sorafenib responder and non-responder samples. Scale bar, 200 and 500 μm as indicated. Right panel: quantification of collagen I, α-SMA, Masson’s trichrome, and Picrosirius red area between sorafenib responder and non-responder samples. **B** Comparison of the ECM stiffness between the sorafenib-responder and non-responder groups by unconfined compression analysis. **C** In cultures with soft (0.5 kPa) or stiff (12 kPa) plates, the cell viability assay was performed to examine the inhibitory effects of sorafenib on the growth of HCC cells. **D** Schematic illustration of the liver orthotopic xenograft model used in the subsequent panels. The mice were administered various treatments, including control PBS, sorafenib (30 mg/kg, three times a week), anti-LOX neutralizing antibodies (3 mg/kg; purified; twice a week), or a combination of sorafenib and anti-LOX. Representative in vivo bioluminescence (**E**) and quantitative analysis of bioluminescence signals (**F**) in the orthotopic xenograft tumors of the indicated groups. **G** Representative images of H&E, collagen I, α-SMA, Masson’s trichrome, and Picrosirius red staining of consecutive pathological sections showing ECM deposition in liver tumors from mice subjected to the indicated treatments; scale bar: 200 μm; **H** quantification of collagen I, α-SMA, Masson’s trichrome, and Picrosirius red area in liver tumors from mice subjected to the indicated treatments. **I** Comparison of the ECM stiffness in the liver tumors from mice subjected to the indicated treatments by unconfined compression analysis. ns not significance, *P < 0.05, **P < 0.01, and ***P < 0.001.

Sorafenib is the first-line therapeutic agent for advanced HCC, with its efficacy in significantly improving patient prognosis [6]. However, HCC patients undergoing Sorafenib treatment develop drug resistance within approximately 6 months [13], rendering them unable to benefit from this treatment, which underscores the critical need to elucidate and overcome sorafenib resistance as a pivotal challenge in improving HCC outcomes. To determine the biomechanical basis of sorafenib resistance in HCC, we performed histochemical and biophysical analyses on matched cohorts of sorafenib responders (*n* = 15) and non-responders (*n* = 15). Histomorphometric analysis revealed markedly elevated overall collagen matrix abundance in non-responders compared to responders (Fig. 2A). Unconfined compression analysis demonstrated an increase in tissue stiffness in non-responders (Fig. 2B). To mechanistically link ECM stiffening to therapeutic resistance, we cultured HCC cells on type I collagen-coated matrices with elastic modulus levels of 12 kPa (stiff) and 0.5 kPa (soft), which reflect the natural elasticities of HCC tissues [14]. HCC cells on stiff substrates exhibited significantly reduced sorafenib sensitivity (Fig. 2C). To validate these findings in vivo, we established orthotopic HCC xenografts in C57BL/6 mice and systemically administered a monoclonal lysyl oxidase (LOX)-neutralizing antibody targeting extracellular LOX activity (Fig. 2D). LOX family proteins catalyze the first step of the covalent cross-linking of ECM components, including collagens and elastin [15]. Considering that intracellular LOX activity contributes to tumor progression, we developed a LOX-blocking antibody that specifically targets extracellular LOX activity. This intervention reduced intra-tumoral collagen matrix abundance and decreased tissue stiffness (Fig. 2G–I). Importantly, LOX inhibition synergized with Sorafenib, reducing tumor volume compared to sorafenib monotherapy (Fig. 2E, F). These findings indicated that increased ECM stiffness enhances resistance to sorafenib in HCC.

### ECM stiffness increases G6PD expression

**Fig. 3:**
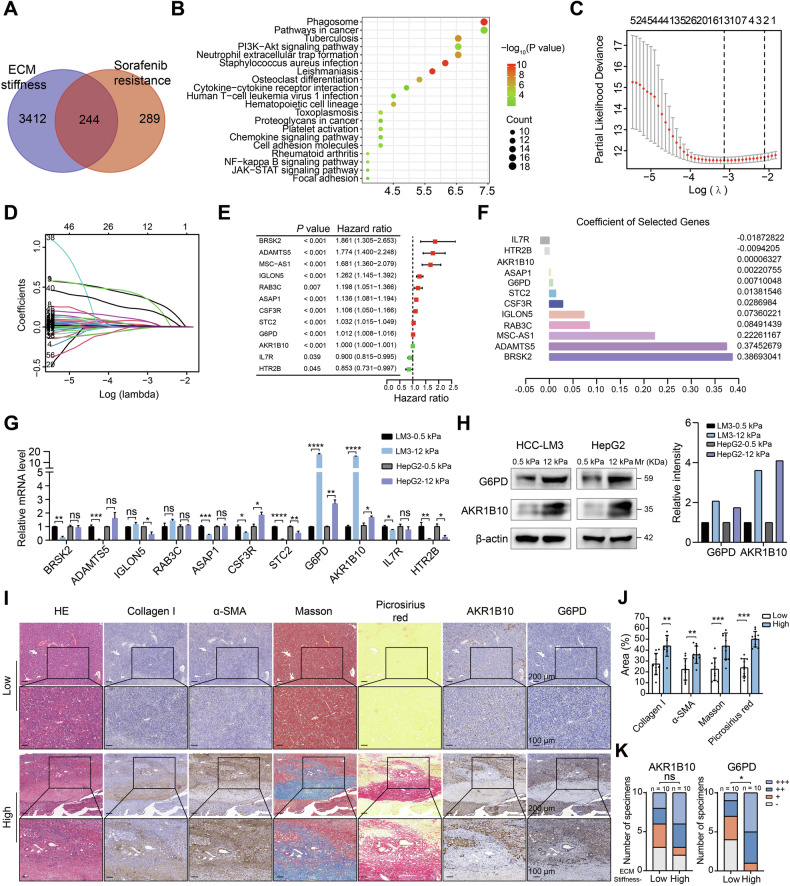
ECM stiffness regulates G6PD expression. **A** Venn diagram illustrating the overlap between genes related to ECM stiffness and those associated with sorafenib resistance. **B** Analysis of the top 20 KEGG pathways for genes related to ECM stiffness and sorafenib resistance. **C** Cross-validation plot for penalty terms. The cross-validation was used to select the optimum value of the regularization parameter (*λ*) in the LASSO regression model. **D** LASSO coefficient profiles of genes associated with ECM stiffness and sorafenib resistance. A coefficient profile plot was produced against the log(*λ*) sequence. A vertical line was drawn at the selected optimal parameter (*λ*), which resulted in 12 nonzero coefficients. **E** A forest plot of 12 genes associated with ECM stiffness and sorafenib resistance. **F** Coefficients of these 12 genes. **G** Real-time qPCR analysis of gene expression related to ECM stiffness under soft (0.5 kPa) or stiff (12 kPa) matrix conditions. **H** Western blotting analysis of G6PD and AKR1B20 expression under soft (0.5 kPa) or stiff (12 kPa) matrix conditions. β-actin was used as a loading control and quantification data were shown on the right panel. **I** Representative images of H&E, Collagen I, α-SMA, Masson’s trichrome, Picrosirius red, AKR1B10, and G6PD staining in HCC tissues from patients who exhibited low stiffness and responsiveness to sorafenib and those with high stiffness and resistance to sorafenib. Scale bar, 200 and 500 μm as indicated. **J** Quantification of collagen I, α-SMA, Masson’s trichrome, and Picrosirius red area between 10 sorafenib-resistant samples with high ECM and 10 sorafenib-sensitive samples. **K** Association between AKR1B10 and G6PD expression levels and the degree of ECM stiffness in HCC tissue. ns not significance; *P < 0.05, **P < 0.01, ***P < 0.001, and ****P < 0.0001.

To elucidate the molecular mechanisms underlying ECM stiffness and sorafenib resistance in HCC, we used the GEO database (GSE143233), which contains transcriptome data from sorafenib-resistant and sorafenib-sensitive patients. Differential expression analysis (FC > 2, *P* < 0.05) identified 533 sorafenib resistance-associated genes, with cross-referencing against 3656 previously identified ECM stiffness-related DEGs, yielding 244 overlapping candidates (Fig. 3A). Kyoto Encyclopedia of Genes and Genomes (KEGG) pathway enrichment of these overlapping genes revealed interesting results, including matrix-related “cell adhesion molecules” and “focal adhesion”; immune-related “neutrophil extracellular trap formation” and “human T-cell leukemia virus 1 infection”; “PI3K-AKT signaling pathway”; “NK-κB signaling pathway”; and “JAK-STAT signaling pathway” (Fig. 3B). Univariate Cox regression and least absolute shrinkage and selection operator (LASSO) regression analyses were performed to identify an ECM stiffness-related and sorafenib resistance-related risk prognostic signature (Fig. 3C, D). Additionally, univariate and multivariate Cox regression analyses were conducted to assess the independent prognostic value of 12 genes for OS (Fig. 3E, F), and a Kaplan–Meier plot was generated to display the differences in the survival times associated with the 12 genes in the TCGA database (Supplementary Fig. 2). Functional validation using HCC-LM3 and HepG2 cells cultured on soft (0.5 kPa) and stiff (12 kPa) plates demonstrated stiffness-dependent upregulation of G6PD and AKR1B10 at mRNA level (Fig. 3G). Western blotting quantification confirmed corresponding protein overexpression under stiff conditions (Fig. 3H). To determine the relationship between G6PD and AKR1B10 and ECM stiffness in HCC, we performed immunohistochemical (IHC) validation in a matched cohort stratified by unconfined compression analysis-measured matrix stiffness and therapeutic response: 10 sorafenib-resistant patients with high ECM and 10 sorafenib-sensitive patients with low stiffness. G6PD expression strongly correlated with ECM stiffening in sorafenib-resistant HCC patients, while AKR1B10 showed no association (Fig. 3I–K). Therefore, G6PD was selected for further investigation.

### G6PD plays an oncogenic role in HCC

To interrogate the functional role of G6PD in HCC progression, we generated stable G6PD-knockdown HCC-LM3 and HepG2 cells using lentiviral shRNA constructs (sh1 and sh2). Knockdown efficiency was using quantitative real-time reverse transcription polymerase chain reaction (qRT-PCR) and Western blotting analyses (Supplementary Figs. 3A and 2B).

**Fig. 4:**
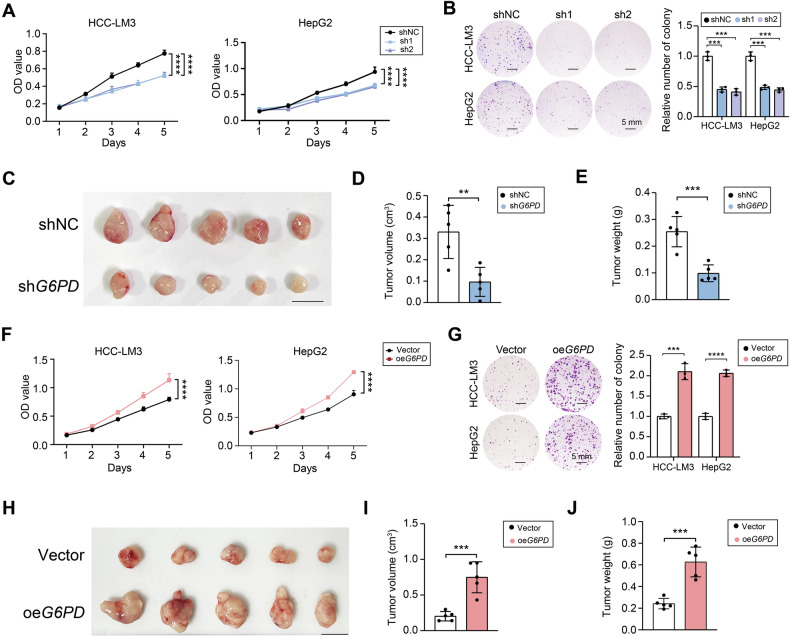
G6PD plays a crucial role in the proliferation of HCC. **A** Comparison of the viability of HCC-LM3 and HepG2 cells between the control group and the G6PD-silenced group. **B** Effects of knocking down G6PD on the growth of HCC-LM3 and HepG2 cells, as determined by plate colony formation assays. **C** The effect of knocking down G6PD on subcutaneous xenograft growth. Statistical analysis of subcutaneous tumor weight (**D**) and tumor volume (**E**) in the control and G6PD-knockdown groups; scale bar: 1 cm. **F** The effect of overexpressing G6PD on the growth of HCC-LM3 and HepG2 cells was evaluated via a CCK8 assay. **G** A plate colony formation assay revealed the effect of overexpressing G6PD on HCC-LM3 and HepG2 cell growth. **H** Growth of subcutaneous tumors in the control group and the G6PD-overexpression group. Statistical analysis of subcutaneous tumor weight (**I**) and tumor volume (**J**) in the control groups and the G6PD-overexpression groups; scale bar: 1 cm; **P < 0.01, ***P < 0.001, and ****P < 0.0001.

Next, CCK8 and colony formation assays were performed, and the results revealed that G6PD knockdown significantly inhibited the proliferation of HCC cells (Fig. 4A, B). In vivo validation using subcutaneous xenografts model, G6PD knockdown reduced tumor volume and tumor weight compared to controls (Fig. 4C–E). Complementary gain-of-function studies utilizing lentiviral G6PD overexpression (oe*G6PD*) enhanced HCC cell proliferation in vitro and accelerated xenograft growth (Fig. 4F–J). These bidirectional experiments indicated that G6PD might act as an oncogene in HCC by promoting the proliferation of HCC.

### ECM stiffness increases sorafenib resistance in HCC via ITGB1-G6PD regulation

**Fig. 5:**
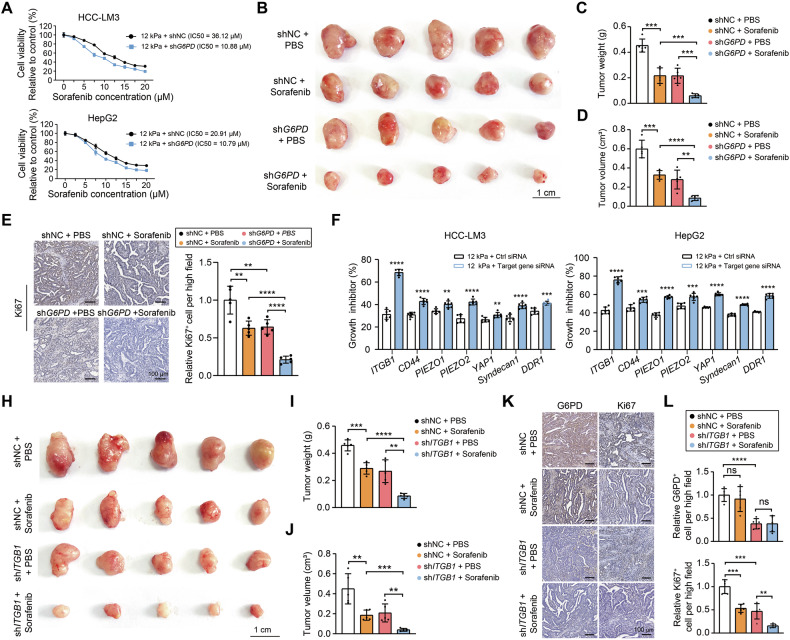
ECM stiffness facilitates resistance to sorafenib through the upregulation of G6PD expression. **A** IC50 curves of shNC and sh*G6PD* HCC-LM3 and HepG2 cells treated with different concentrations of sorafenib (0, 5, 10, 15, or 20 μM) in a 12 kPa matrix culture. **B** Subcutaneous tumors inoculated with shNC or sh*G6PD* HCC-LM3 cells treated with sorafenib. **C**, **D** Statistical analysis of tumor weight and volume in the control, sorafenib-treated, sh*G6PD*-treated, and shG6PD + sorafenib-treated groups. **E** IHC analysis of Ki67 expression in the indicated treatment groups; scale bar: 100 μm. Right panel: quantification of Ki67-positive cell in the indicated treatment groups. **F** Effects of sorafenib treatment on the growth of HCC-LM3 and HepG2 cells under 12 kPa matrix culture conditions in the presence or absence of siRNAs targeting responsive receptors. **H** Growth of subcutaneous tumors inoculated with shNC or sh*ITGB1* HCC-LM3 cells treated with sorafenib. **I**, **J** Statistical analysis of tumor weight and volume in the control, sorafenib, sh*ITGB1*, and sh*ITGB1* + sorafenib treatment groups. **K** IHC analysis of G6PD (left panel) and Ki67 expression (right panel) in the indicated groups. Scale bar: 100 μm. **L** Quantification of G6PD-positive cell (upper panel) and Ki67-positive cell (lower panel) in the indicated treatment groups. ns not significance, **P < 0.01, ***P < 0.001, and ****P < 0.0001.

To investigate the role of G6PD in the ability of ECM stiffness confers sorafenib resistance in HCC, sorafenib IC50 assays were performed. The results revealed that promoting sorafenib resistance in HCC cells caused by an increase in ECM stiffness was partially eliminated by knocking down G6PD (Fig. 5A). In a subcutaneous xenograft model, the results confirmed that G6PD silencing synergized with sorafenib reducing tumor weight and tumor volume compared to sorafenib monotherapy (Fig. 5B–D). IHC staining for Ki67 confirmed that G6PD knocking down significantly increased the inhibitory effect of sorafenib on the proliferation of HCC cells (Fig. 5E). As ECM stiffness generally regulates tumor progression through its receptors [16], we systematically screened several common ECM-sensing receptors (ITGB1, CD44, Piezo1, Piezo2, YAP1, Syndecan1, and DDR1) via siRNA knockdown, and confirmed the knockdown efficiency by conducting qRT-PCR analyses (Supplementary Fig. 3E). Next, the HCC cells were cultured on plates with different stiffness values (0.5 kPa and 12 kPa), and the inhibitory effect of sorafenib on cell proliferation was assessed via CCK8 assays. The results indicated that ITGB1 knockdown significantly inhibited the reduction in sorafenib sensitivity caused by an increase in ECM stiffness (Fig. 5F). The in vivo validation using subcutaneous xenografts demonstrated that sorafenib significantly reduced tumor volume and weight under ITGB1 silencing compared to controls (Fig. 5H–J), with parallel suppression of Ki67+ proliferative cells (Fig. 5K, L). Additionally, ITGB1 knockdown abrogated the expression of G6PD in tumor cells (Fig. 5K, L). These findings suggested that G6PD promotes the effect of ECM stiffness on sorafenib resistance in HCC and that ITGB1, a receptor that senses ECM stiffness, is involved in the regulation of sorafenib resistance.

### ITGB1 modulates G6PD expression through the PI3K/AKT pathway

**Fig. 6:**
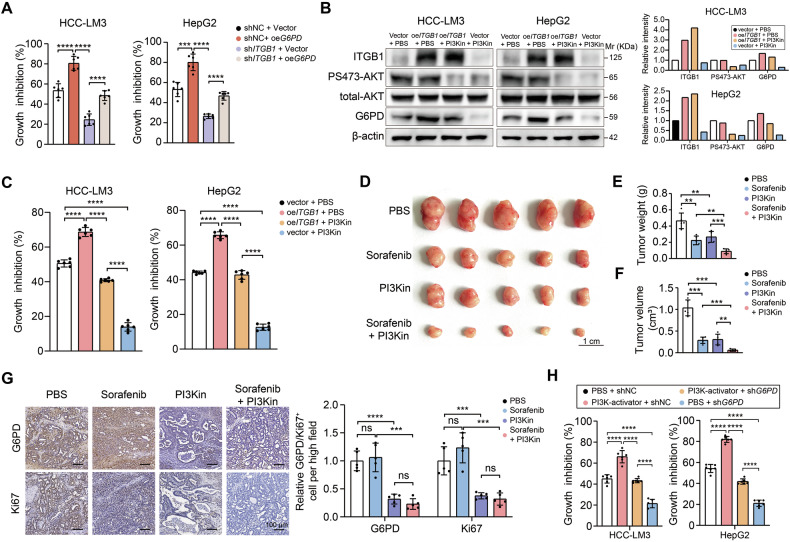
ITGB1 regulates G6PD expression and sorafenib resistance through the PI3K-AKT pathway. **A** Effect of ITGB1 silencing on the resistance of vector or oe*G6PD* HCC-LM3 and HepG2 cells to sorafenib, as determined by CCK8 assays. **B** Effects of ITGB1 overexpression in combination with the PI3K inhibitor LY294002 on the expression levels of pS473-AKT and G6PD in HCC-LM3 and HepG2 cells, as determined by Western blotting assays. β-actin was used as a loading control, and quantification data were shown on the right panel. **C** Viability of ITGB1-overexpressing HCC-LM3 and HepG2 cells treated with sorafenib in the presence or absence of the PI3K inhibitor LY294002. **D** Subcutaneous tumor growth was monitored under sorafenib treatment in the control, sorafenib, PI3K inhibitor (LY294002), and sorafenib + LY294002 groups. **E**, **F** Tumor weight and volume were analyzed in the control, sorafenib treatment, PI3K inhibitor (LY294002) treatment, and sorafenib + LY294002 groups. **G** IHC staining for G6PD (upper panel) and Ki67 (lower panel) was conducted in the indicated groups; scale bar: 100 μm. Right panel: quantification of G6PD and Ki67-positive cell in the indicated treatment groups. **H** Cell viability experiments were performed to assess the inhibitory effects of sorafenib on HCC-LM3 and HepG2 cell growth in the control, PI3K agonist (740 Y-P), G6PD knockdown, and G6PD knockdown + PI3K agonist treatment groups; **P < 0.01, ***P < 0.001, and ****P < 0.0001.

To interrogate the mechanistic hierarchy between ITGB1-mediated mechano-signaling and G6PD-driven sorafenib resistance in HCC, stable ITGB1-knockdown models were generated in G6PD-overexpressing (oe*G6PD*) HCC-LM3 and HepG2 cell lines. Pharmacodynamic profiling via CCK-8 assays demonstrated that G6PD overexpression partially restored sorafenib tolerance in ITGB1-depleted cells, suggesting G6PD operates downstream of ITGB1 in mediating sorafenib resistance (Fig. 6A). Cross-database interrogation (TCGA/GSE143233) identified 244 ECM stiffness-sorafenib resistance coregulated genes, with KEGG pathway analysis revealing pronounced enrichment in PI3K-AKT signaling (Fig. 3B). As G6PD is a key enzyme in pentose phosphate pathway (PPP) metabolism and can regulate cancer metabolism [17], we investigated the relationship between G6PD and the PI3K/AKT pathway, which plays an important role in cancer metabolism [18]. G6PD expression, which was upregulated by ITGB1 overexpression, was significantly decreased by the PI3K inhibitor LY294002 (Fig. 6B). Growth inhibition experiments confirmed that the effect of ITGB1 overexpression on enhancing sorafenib tolerance in HCC cells could be offset by the PI3K inhibitor (Fig. 6C). To assess the effect of the PI3K inhibitor on sorafenib resistance in vivo, a subcutaneous xenograft model was used, and the results revealed that PI3K inhibition synergized with sorafenib, reducing tumor weight, tumor volume (Fig. 6D–F) and Ki67 proliferation index, with parallel G6PD suppression versus monotherapy (Fig. 6G). Growth inhibition experiments revealed that a PI3K agonist (740Y-P) increased the resistance of HCC cells to sorafenib and that G6PD silencing partly reversed this effect (Fig. 6H). These results suggested that the PI3K-AKT pathway mediates the regulatory effect of ITGB1 on G6PD expression, which also affects sorafenib resistance in HCC cells.

### Clinical relevance of the ECM stiffness-ITGB1-PI3K/AKT-G6PD axis and sorafenib resistance in HCC

**Fig. 7:**
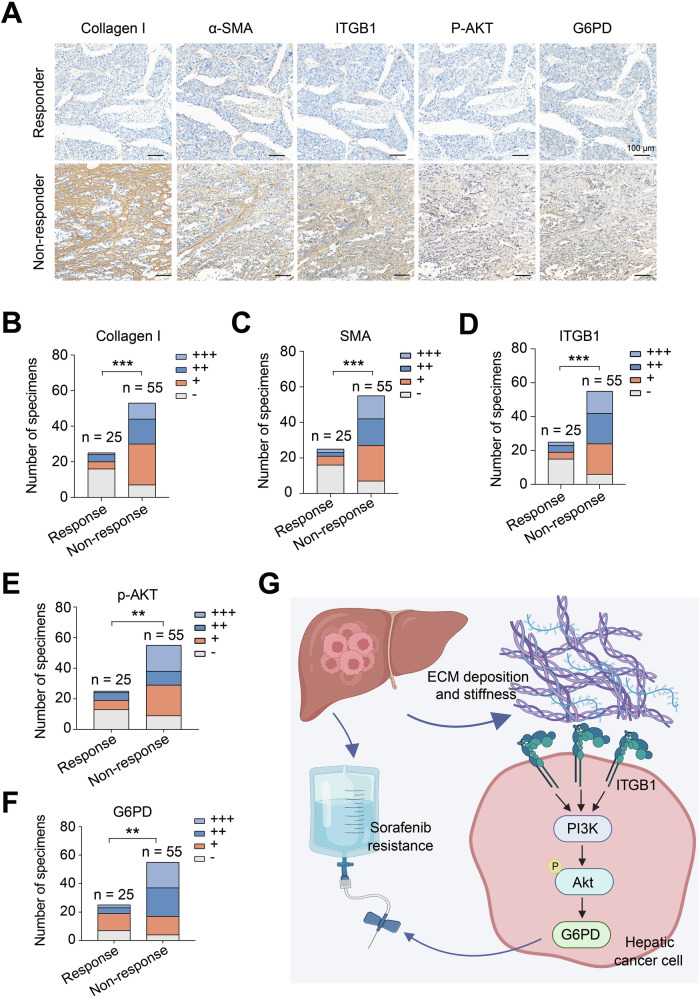
Clinical relevance of the ECM stiffness-ITGB1-PI3K/AKT-G6PD axis and sorafenib resistance in HCC. **A** Representative IHC images of collagen I, α-SMA, ITGB1, p-AKT, and G6PD in HCC tissue microarrays (*n* = 80). Correlations between the expression levels of Collagen I (**B**), α-SMA (**C**), ITGB1 (**D**), p-AKT (**E**), and G6PD (**F**) in the HCC cohort. **G** Schematic diagram illustrating the mechanism by which ECM stiffness regulates sorafenib resistance in HCC patients; **P < 0.01, and ***P < 0.001.

To comprehensively understand the relationships among ECM stiffness, ITGB1, p-AKT, G6PD, and sorafenib resistance in HCC, we studied a cohort of 80 patients with HCC. The expression levels of ITGB1, G6PD, p-AKT, and ECM stiffness-related proteins (collagen I and α-SMA) were determined via IHC assays (Fig. 7A). Compared to the Sorafenib responder samples, the non-responder samples presented higher expression levels of ECM stiffness-related proteins (Fig. 7B, C), ITGB1 (Fig. 7D), p-AKT (Fig. 7E), and G6PD (Fig. 7F). Overall, our study revealed that G6PD, which signals downstream of ECM stiffness, plays a crucial role in sorafenib resistance, and this role is mediated by the ECM-sensing receptor ITGB1 through the activation of the PI3K/AKT pathway (Fig. 7G).

## Discussion

Sorafenib is used as the standard first-line therapeutic agent for patients with advanced HCC. Clinical studies have demonstrated that sorafenib can considerably extend the median survival time in advanced HCC patients. A phase III clinical trial has also verified the non-inferiority of lenvatinib compared to sorafenib in terms of OS in untreated advanced HCC patients. Besides sorafenib, regorafenib, cabozantinib, and ramucirumab have gained approval as secondary treatment options [19–22]. However, the prevalence of de novo resistance to sorafenib among HCC patients is considerable, with only about 35% experiencing therapeutic benefits. Additionally, patients who initially respond to sorafenib often develop resistance within six months [7, 23]. The mechanisms of resistance to sorafenib need to be elucidated. Our study revealed that ECM stiffness is a critical regulatory mechanism in sorafenib resistance. Inhibition of ECM stiffness significantly enhances the therapeutic effect of sorafenib on HCC. Moreover, the degree of ECM stiffness in tissue samples from HCC patients is closely associated with resistance to sorafenib.

Considering the role of ECM stiffness as a high-risk factor in various cancers and its strong association with tumor progression, it represents a highly attractive therapeutic target in cancer. ECM stiffness contributes to the regulation of various biological processes through the provision of mechanical cues, including epithelial-to-mesenchymal transition, autophagy, and metabolic reprogramming [16, 24–26]. A comprehensive understanding of the mechanisms by which ECM stiffness drives tumor progression and resistance to chemotherapy is essential for developing targeted therapy for cancer. Studies have indicated that ECM stiffness can increase paclitaxel resistance in pancreatic cancer and decrease the sensitivity of HCC and ovarian cancer to platinum-based treatments [14, 27]. Moreover, ECM stiffness can increase the sensitivity of tumor cells to genotoxic agents by activating MAPK4/6/7 and increasing ubiquitin phosphorylation, thereby inhibiting ubiquitin signaling and DNA repair following DNA double-strand breaks [28]. However, the relationship between ECM stiffness and sorafenib resistance has not been investigated extensively. We analyzed HCC patients from the TCGA database via Euclidean distance-based unsupervised hierarchical clustering and found a significant correlation between ECM stiffness and drug resistance. Subsequent in vitro and in vivo experiments confirmed that ECM stiffness reduces the sorafenib sensitivity of HCC cells. To elucidate the mechanisms by which ECM stiffness regulates sorafenib resistance, we analyzed sorafenib resistance-related DEGs in the GEO database and identified G6PD as a potential driver of sorafenib resistance regulated by ECM stiffness.

G6PD, which acts as the rate-limiting enzyme in the pentose phosphate pathway, can modulate multiple oncogenic processes through metabolic reprogramming and redox homeostasis alterations [29]. Recent studies have associated aberrant G6PD activity with critical cancer-related phenomena, such as transformation, metastasis, angiogenesis, and resistance to therapy, in several types of cancer [10]. Endogenous reductants such as NADPH and GSH produced by G6PD play an important role in determining the drug sensitivity of tumor cells under stress. Knocking out G6PD protects colorectal cancer cells from oxaliplatin-induced apoptosis [30]. In patient-derived xenograft models, the silencing of G6PD can increase the sensitivity of colorectal cancer cells to oxaliplatin [30]. Additionally, G6PD overexpression, which leads to lactate accumulation and the formation of an acidic microenvironment, has been shown to increase tumor cell tolerance to chemotherapy [31]. Consistent with these findings, our study revealed that the combination of G6PD silencing with sorafenib significantly increased the efficacy of the inhibitory effects of sorafenib on HCC cell proliferation. Additionally, tissue microarray analysis revealed a correlation between G6PD expression levels and the response to sorafenib in HCC patients. These findings suggested that G6PD can serve as a therapeutic target to overcome sorafenib resistance.

The transmembrane glycoprotein signaling receptor ITGB1 mediates the tumor-promoting effects of ECM stiffness by transmitting mechanical signals. ITGB1 regulates tumor cell resistance through downstream signaling pathways [32]. For example, in pancreatic cancer, the ITGB1-driven Src-AKT pathway, which is independent of EGFR signaling, enhances resistance to cetuximab therapy [33]. ITGB1 also potentiates PDAC resistance to gemcitabine by activating CDC42 in the PI3K-p110β signaling pathway [34]. However, the regulatory role of ITGB1 in G6PD and the underlying mechanism remain unknown. Our study established that ITGB1 mediates ECM stiffness-driven sorafenib resistance through PI3K/AKT pathway activation, subsequently upregulating G6PD expression in vitro. In vivo validation demonstrated that ITGB1 knockdown combined with sorafenib treatment significantly enhances the cytotoxic effects of sorafenib on HCC cells. These findings suggest that targeting the ITGB1-PI3K/AKT-G6PD axis may be a suitable strategy to counteract the influence of ECM stiffness on sorafenib resistance in HCC.

To summarize, ECM stiffness is significantly associated with unfavorable clinical outcomes and responsiveness to sorafenib therapy in patients with HCC. Moreover, the ITGB1-PI3K/AKT-G6PD axis is implicated in the regulatory mechanism of sorafenib resistance mediated by ECM stiffness. Our findings provided novel theoretical perspectives and potential therapeutic targets for HCC patients resistant to sorafenib.

## Methods

### Ethical approval

The human tissue samples used for IHC staining were obtained from HCC patients who were receiving sorafenib treatment at the Affiliated Hospital of Qingdao University. The samples were divided into responder and non-responder groups based on the mRECIST criteria. The HCC specimens subjected to IHC analysis in this study were procured from treatment-naive HCC patients with a clinically confirmed absence of cirrhotic etiology. The study was approved by the research ethics committee of the Affiliated Hospital of Qingdao University, Qingdao, China (Identification code: QYFY WZLL 29040). Informed consent was obtained from all patients.

### Cell lines and drug information

The human liver cancer cell lines (HCC-LM3, SNU-389, Huh-7, Hep3B, SMMC-7721, SNU-475, HepG2, MHCC-97 L, and SK-Hep-1) and the mouse liver cancer cell lines (Hepa1-6) were obtained from the Chinese Academy of Sciences (Shanghai, China) or American Type Culture Collection (ATCC, Manassas, VA, USA). The cells were maintained in a culture medium as suggested by the ATCC protocol, supplemented with 10% fetal bovine serum (FBS; Gibco, USA) and 1% streptomycin-penicillin (Sigma-Aldrich, Shanghai, China) at 37 °C in a 5% CO_2_ environment. Sorafenib (S7397), LY294002 (S1105, 20 μM), and 740 Y-P (S7865, 15 µM) were purchased from Selleck (Shanghai, China). The anti-LOX function-blocking antibody was purchased from SinoBiological (Shanghai, China) and administered at a dose of 3 mg/kg, twice a week.

### Colony formation assay

Cells obtained from a single specific group of HCC cells were plated in a 6-well plate at a density of 1000 cells per well, and the culture medium was changed every 2 days. After 2 weeks, the cell colonies were fixed with a 4% paraformaldehyde solution and then stained with 0.1% crystal violet. The colonies, each containing more than 50 cells, were subsequently counted under a microscope.

### IC50 analysis

HCC cells subjected to specific treatments were plated in a 96-well plate (12 kPa, Irvine, CA, USA) at a density of 10,000 cells per well. After 24 h, a complete medium containing different concentrations of sorafenib (0, 5, 10, 15, or 20 μM) was added to each well. After 48 h, a CCK-8 reagent (10 μL/well, CCK-8, Dojindo, Japan) mixed with serum-free medium (90 μL/well) was added to each well. The cells were then incubated at 37 °C for 1 h. The absorbance was measured at 450 nm using a Power Wave XS microplate reader (BIO-TEK), with a reference wavelength of 600 nm. The viability was calculated using the following formula: cell viability (%) = (treatment group/control group) × 100%.

### RNA extraction and qRT-PCR

Total RNA was extracted from frozen tissues or cells using TRIzol reagent and reverse transcribed following the instructions of the PrimeScript RT-PCR Kit. Real-time qRT-PCR analysis was performed on the ViiA7 real-time PCR system using SYBR Green Premix Ex Taq (B21203, Bimake, China). Relative mRNA expression was calculated using the 2^−ΔΔCt^ method, and the mRNA expression level of 18S was used as a control for analysis. The primer sequences used in this study are listed below.

**Table:** 

Gene	Forward primer (5’→3’)	Reverse primer (5’→3’)
G6PD	CGAGGCCGTCACCAAGAAC	GTAGTGGTCGATGCGGTAGA
ITGB1	CCTACTTCTGCACGATGTGATG	CCTTTGCTACGGTTGGTTACATT
CD44	CTGCCGCTTTGCAGGTGTA	CATTGTGGGCAAGGTGCTATT
PIEZO1	GGACTCTCGCTGGTCTACCT	GGGCACAATATGCAGGCAGA
PIEZO2	ATGGCCTCAGAAGTGGTGTG	ATGTCCTTGCATCGTCGTTTT
YAP1	TAGCCCTGCGTAGCCAGTTA	TCATGCTTAGTCCACTGTCTGT
Syndecan1	CCACCATGAGACCTCAACCC	GCCACTACAGCCGTATTCTCC
DDR1	CCGACTGGTTCGCTTCTACC	CGGTGTAAGACAGGAGTCCATC
18S	TGCGAGTACTCAACACCAACA	GCATATCTTCGGCCCACA

### Cell transfection

Before transfection, HCC cells were seeded in six-well plates. After 24 h, cells at 60–70% density were transfected with 50 ng of specific small interfering RNAs (siRNAs) targeting ITGB1, CD44, PIEZO1, PIEZO2, PAR1, Syndecan1, or DDR1 or with siRNAs not targeting any known gene sequences. The transfection was performed following the instructions provided with the Lipofectamine 481® RNAiMAX reagent (Thermo Fisher Scientific, #1378030). The siRNA oligonucleotides were synthesized by GenePharma (Shanghai, China). The siRNA sequences used in this study were as follows: si-*G6PD*-1: GCCUUCCAUCAGUCGGAUATT; si-*G6PD*-2: GAGAGUGGGUUUCCAGUAUTT; si-*G6PD*-3: GCGUUAUCCUCACCUUCAATT; si-*ITGB1*: GCCUUGCAUUACUGCUGAUTT; si-*CD44*: GCCCUAUUAGUGAUUUCCATT; si-*PIEZO1*: CUCACCAAGAAGUACAAUCTT; si-*PIEZO2*: CCAGAGACAAUACAACUAATT; si-*DDR1*: GUAUUUAUCUGAGGCCGUGTT; si-*Syndecan1*: CCGACUGCUUUGGACCUAATT; si-*YAP1*: CCCAGUUAAAUGUUCACCATT. The knockout efficiency was determined 48 h after transfection via qRT-PCR analysis, and the cells were harvested or processed for further experiments.

### Generation of shRNA cell lines

The specific shRNA oligonucleotides targeting G6PD were synthesized by GenePharma (Shanghai, China). The shRNAs used included sh*-G6PD*: CAACAGATACAAGAACGTGAA; sh-*ITGB1*: GCCTTGCATTACTGCTGATAT. Before transfection, HCC cells were seeded in six-well plates. When the cells reached 60–70% confluence, a mixture of 10 μL/well lentivirus suspension and 1 mL/well culture medium containing 5 μg/mL polybrene (Gene Pharma, Shanghai, China) was added to each well. Puromycin (5 μg/mL) was added to the culture medium to select cells that stably expressed the shRNA.

### Western blotting analysis

Total cellular proteins were extracted using an IP lysis buffer, and protein concentrations were determined using the Pierce BCA Protein Assay Kit. The proteins were separated by 8–12% sodium dodecyl sulfate–polyacrylamide gel electrophoresis and transferred to polyvinylidene fluoride membranes. The membranes were blocked with 5% (m/v) skim milk in Tris-buffered saline for 1 h and then incubated with specific primary antibodies (4 °C, overnight). The next day, the target proteins were visualized using an Odyssey imaging system with horseradish peroxidase (HRP)-conjugated secondary antibodies (Cell Signaling Technology, China). The primary antibodies used were as follows: G6PD (Proteintech, 25413–1-AP, 1:2000), AKR1B10 (Proteintech, 18252–1-AP, 1:2000), ITGB1 (Proteintech, 12594–1-AP, 1:5000), AKT (Proteintech, 10176–2-AP, 1:2000), p-AKT (Ser473, Proteintech, 66444–1-Ig, 1:2000), and β-actin (Proteintech, 66009–1-Ig, 1:20,000).

### IHC analysis and tissue microarray

First, tissue sections (5 μm) were routinely deparaffinized and hydrated, followed by citrate-based antigen retrieval and removal of endogenous peroxidase in the tissue using an endogenous peroxidase inhibitor. Then, the tissue sections were blocked with 10% (m/v) BSA for 1 h at room temperature. Next, the membranes were incubated with primary antibodies overnight at 4 °C. The next day, the sections were incubated with HRP-conjugated secondary antibodies (Cell Signaling Technology, China) for 1 h at room temperature. Finally, immunoreactivity was observed with DAB, and the sections were counterstained with hematoxylin. All tissue sections were photographed under a microscope. The primary antibodies used were as follows: G6PD (Proteintech, 25413–1-AP, 1:500), AKR1B10 (Proteintech, 18252–1-AP, 1:2000), ITGB1 (Proteintech, 12594–1-AP, 1:1000), collagen I (Proteintech, 10176–2-AP, 1:2500), α-SMA (Proteintech, 66009–1-Ig, 1:500), and p-AKT (Ser473, Proteintech, 66444–1-Ig, 1:2000).

### Elastic modulus measurements

Biomechanical characterization of HCC tissues was performed through unconfined compression analysis using a Mach-1 mechanical analyzer (v500c; Biomomentum Inc., Canada) equipped with a 1.5 N load cell (resolution: 0.075 mN) and precision translation stage (positional accuracy: ±0.5 µm). Cuboidal specimens (about 5 × 5 × 5 mm^3^) were excised from fresh tumor samples and hydrated with PBS to maintain physiological hydration. Automated compression was applied at a strain rate of 0.1 mm/s with an amplitude ranging from 1.5 to 2 mm, ensuring measurements remained within the linear viscoelastic regime. The elastic modulus was calculated based on the slope of the linear regression fit to the stress-strain curve, using data obtained from the initial 10% of the maximum.

### ECM score and drug sensitivity analyses

The R package GSVA (Version 1.46.0) was implemented to compute single-sample Gene Set Enrichment Analysis (ssGSEA) scores for the ECM-associated gene set (MMP11, ADAMTS14, SERPINH1, COL10A1, COL11A1, MMP1, COL1A1, LOXL2, MMP9, ADAM12, ACAN, SPP1, FAP, ADAM8, COL5A2, TIMP1, MMP12, ITGAX, COL5A1, COL7A1, COL5A3, TGFBI, COMP, MFAP2, VCAN, COL1A2, COL3A1, SULF1, POSTN, FN1) using transcriptomic profiles of individual specimens. Thereafter, samples were stratified into high- and low-scoring groups based on median-centered ssGSEA score distribution to facilitate downstream investigations [35]. All drug resistance-related data were obtained from the Cancer Cell Line Encyclopedia and the GDSC database. An ECM score was considered to be associated with drug resistance if the following criteria were met: |R| > 0.3 and *P* < 0.05.

### Establishment and verification of the risk prognostic model

We initially performed univariate Cox regression analysis on the TCGA-HCC dataset to identify genes with prognostic significance (p < 0.05). A risk model was constructed using ECM- and sorafenib resistance-associated genes. Next, we conducted the LASSO regression analysis to further develop and enhance the risk model. The resulting equation was as follows: risk score = Σ[Exp (gene) × Coef (gene)]. Here, Exp (gene) refers to the gene expression level, and coef (gene) represents the regression coefficient.

### In vivo experiments

To construct the orthotopic tumor model, 6~8-week-old C57BL/6J male and female (1:1) mice were used. Initially, the mice were anesthetized with 2% isoflurane, followed by the injection of 5 × 10^6^ Hepa1-6 cells suspended in 25 μL of PBS into the mouse liver tissue. Two weeks after surgery, the mice were randomly divided into groups and treated with PBS, sorafenib (30 mg/kg, three times a week), anti-LOX (3 mg/kg, twice a week), or a combination of sorafenib and anti-LOX. After 3 weeks of treatment, the tumor size in the mice was measured by bioluminescence imaging analysis and quantified with Living Image software version 4.5.3.

For the subcutaneous xenograft model, 6~8-week-old male nude mice were used. Based on the experimental requirements, HCC-LM3 cells were genetically knocked down in advance. Then, 100 μL of PBS containing 2 × 10^6^ HCC-LM3 cells was injected subcutaneously into the backs of the nude mice. When the tumors became visible, the mice were treated with sorafenib (30 mg/kg; three times a week) or a PI3K inhibitor (LY294002; 35 mg/kg; three times a week) for 3 weeks. The tumor size was measured using a Vernier caliper. The formula used to calculate tumor volume was as follows: volume = 1/2 × (length × width^2^).

### Statistical analysis

All data were presented as the mean ± standard deviation (SD). The differences between the two independent groups were determined by conducting two-tailed Student’s *t* tests. All data were statistically analyzed using GraphPad Prism (GraphPad Software Inc., San Diego, CA). Nonparametric Spearman correlation analysis was performed to determine the correlation between the ECM score and pathological TNM stage in HCC. Kaplan–Meier survival curves were analyzed by conducting the log-rank test. All results were considered to be statistically significant at *P* < 0.05 (*P < 0.05, **P < 0.01, and ***P < 0.001).

## Supplementary information

Supplementary Figure

WB Raw data

PCR Raw data

## Data Availability

The databases and analysis performed in this study could be obtained by contacting the corresponding author upon reasonable request.
