# Supplementary material for: Matrix stiffness regulates glucose-6-phosphate dehydrogenase expression to mediate sorafenib resistance in hepatocellular carcinoma through the ITGB1-PI3K/AKT pathway
Source: Cell Death Dis. 2025 Jul 20;16(1):538. doi: 10.1038/s41419-025-07842-3 (PMC12277399; doi:10.1038/s41419-025-07842-3)
Supplement: Supplementary file 1 — Supplementary Figure [file 41419_2025_7842_MOESM1_ESM.pdf]

Supplementary Figure

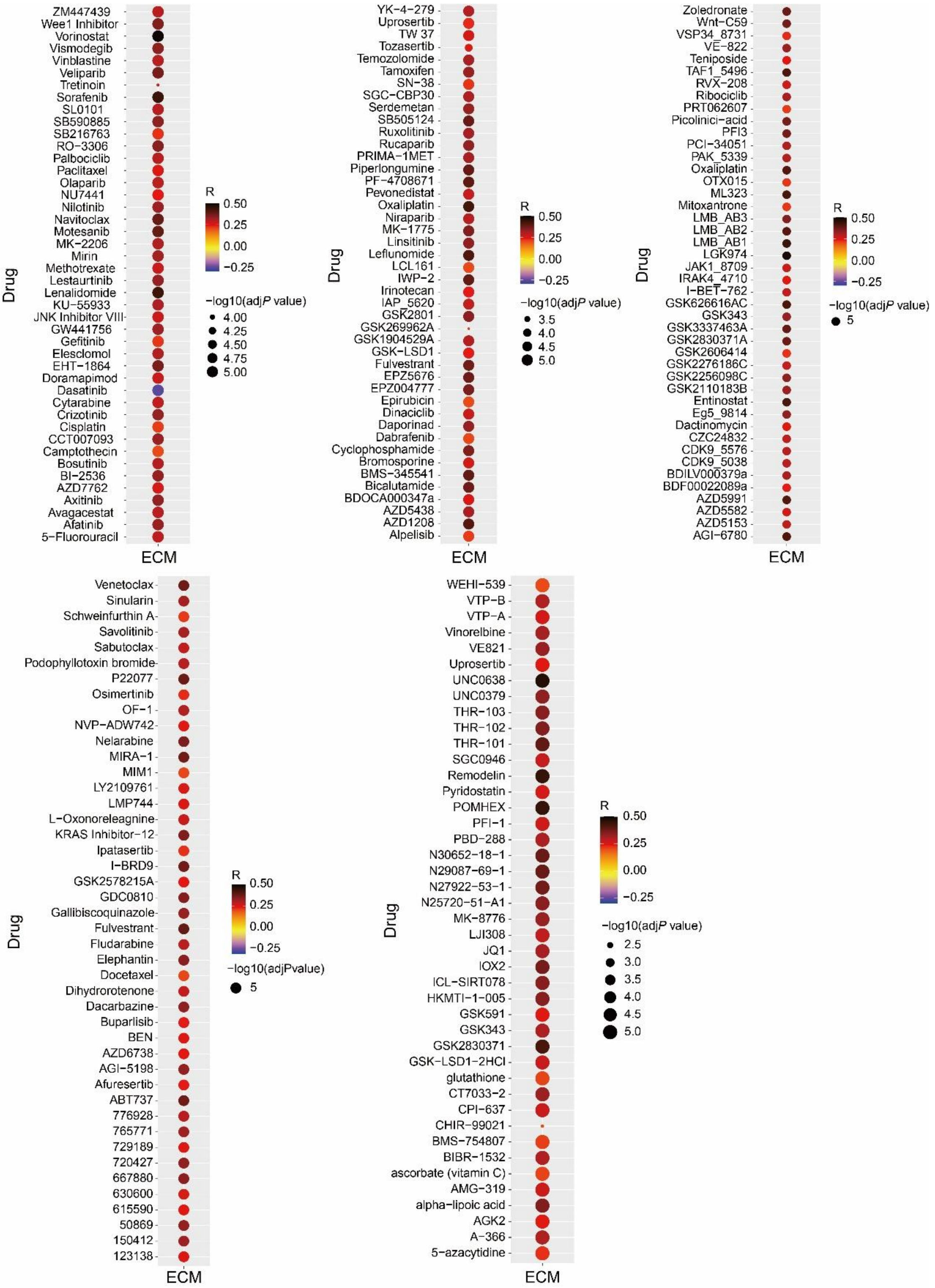

Supplementary Figure 1: Relationships between the ECM score and drug sensitivity according to the Genomics of Drug Sensitivity in Cancer (GDSC) database.

The color gradient in the bubble map represents the R-value. The blank color indicates a positive correlation with sensitivity to the drug candidates, whereas purple indicates a negative

correlation. The size of the nodes represents the statistical significance; the larger the size, the greater the significance.

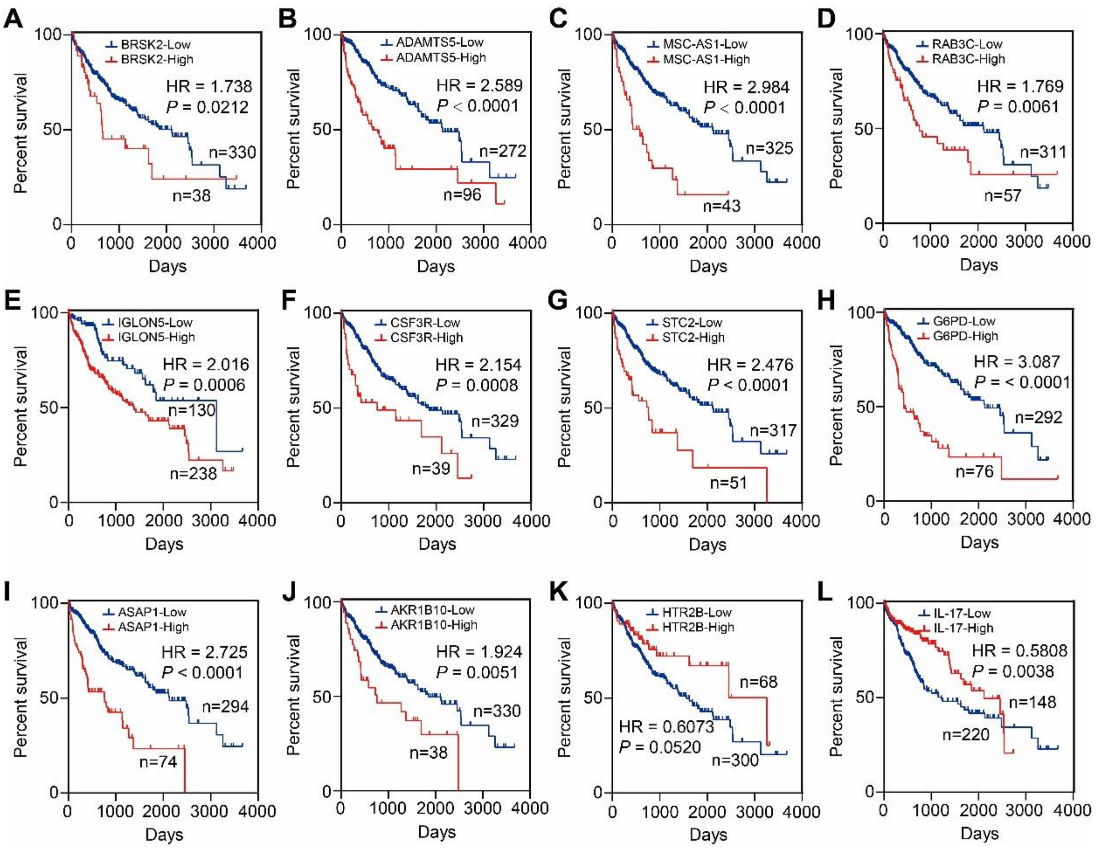

Supplementary Figure 2: Prognostic value of 12 ECM stiffness- and sorafenib resistance-related genes in LIHC

Comparison of Kaplan-Meier survival curves based on high and low levels of BRSK2 (A), ADAMTS5 (B), MSC-AS1 (C), RAB3C (D), IGLON5 (E), CSF3R (F), STC2 (G), G6PD (H), ASAP1 (I), AKR1B20 (J), HTR2B (K), and IL17 (L) in the overall cohort of LIHC patients in TCGA (n = 368) (optimal cutoff).

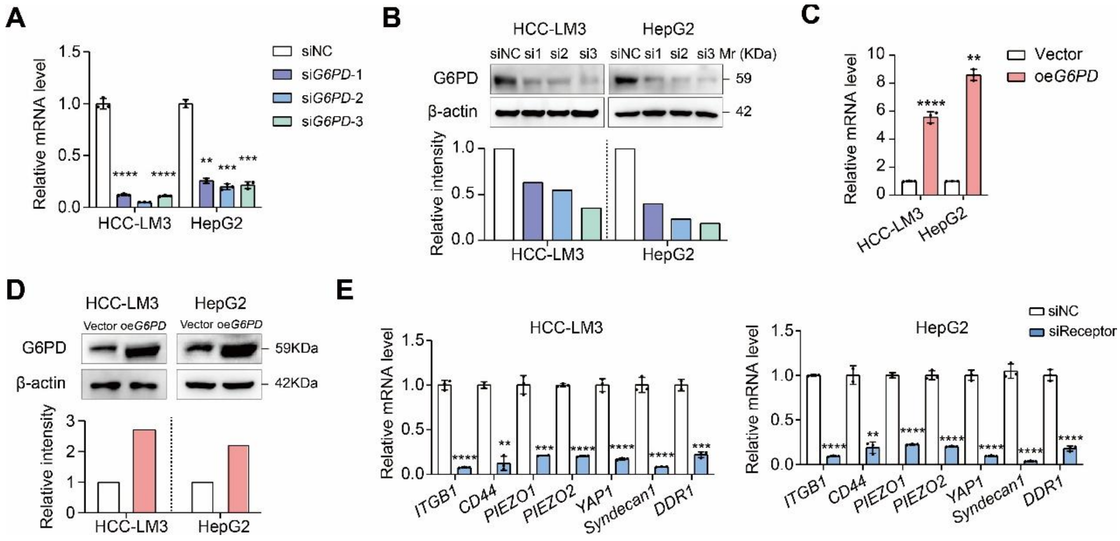

Supplementary Figure 3: (A) qRT-PCR analysis was performed to determine the efficiency of G6PD knockdown in HCC-LM3 and HepG2 cells. (B) Western blotting analysis was conducted to determine the efficiency of G6PD interference in HCC-LM3 and HepG2 cells. β-actin was used as a loading control and quantification data were shown on the lower panel. (C) qRT-PCR analysis was performed to measure the overexpression efficiency of G6PD in HCC-LM3 and HepG2 cells. (D) Western blotting analysis was performed to analyze the overexpression efficiency of G6PD in HCC-LM3 and HepG2 cells. β-actin was used as a loading control and quantification data were shown on the lower panel. (E) qRT-PCR analysis was performed to evaluate the interference efficiency of siRNAs targeting ECM stiffness-sensing receptor genes; $^{**}P < 0.01$, $^{***}P < 0.001$, and $^{****}P < 0.0001$.
