# Supplementary material for: Matrix stiffness regulates glucose-6-phosphate dehydrogenase expression to mediate sorafenib resistance in hepatocellular carcinoma through the ITGB1-PI3K/AKT pathway
Source: Cell Death Dis. 2025 Jul 20;16(1):538. doi: 10.1038/s41419-025-07842-3 (PMC12277399; doi:10.1038/s41419-025-07842-3)

Fig 3H HCC-LM3 G6PD

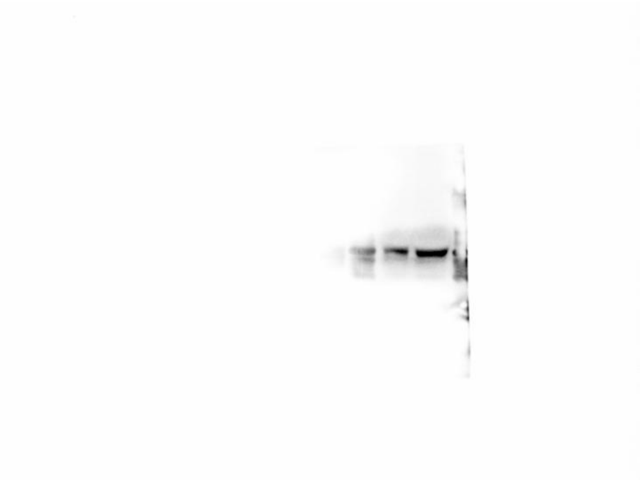

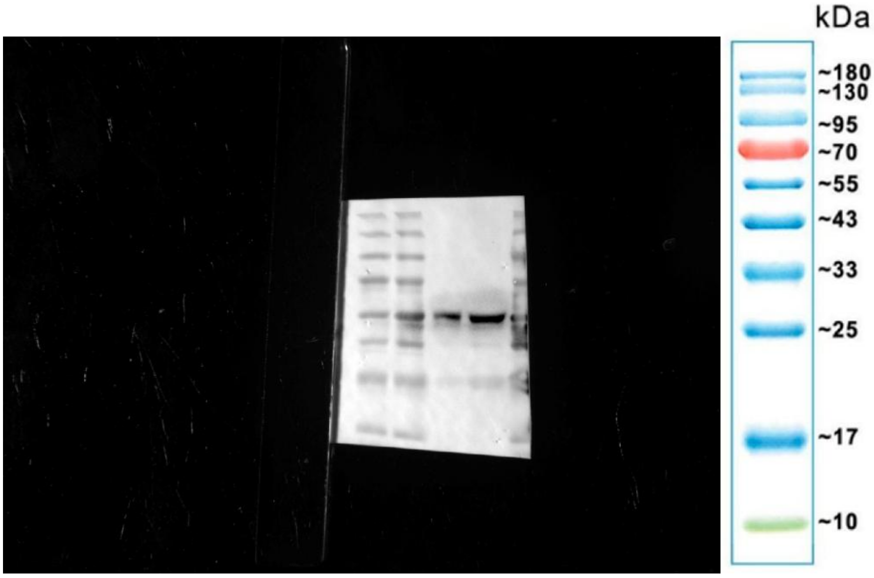

Fig 3H HCC-LM3-AKR1B10

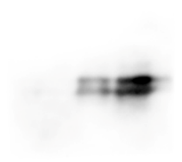

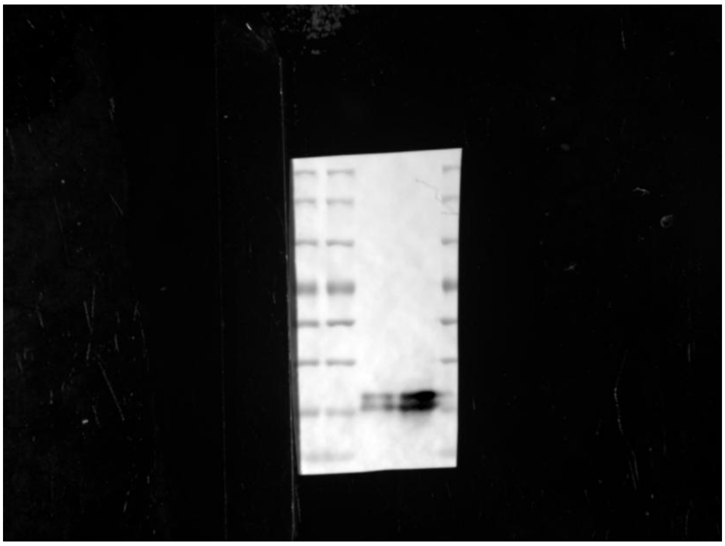

Fig 3H HCC-LM3 Actin

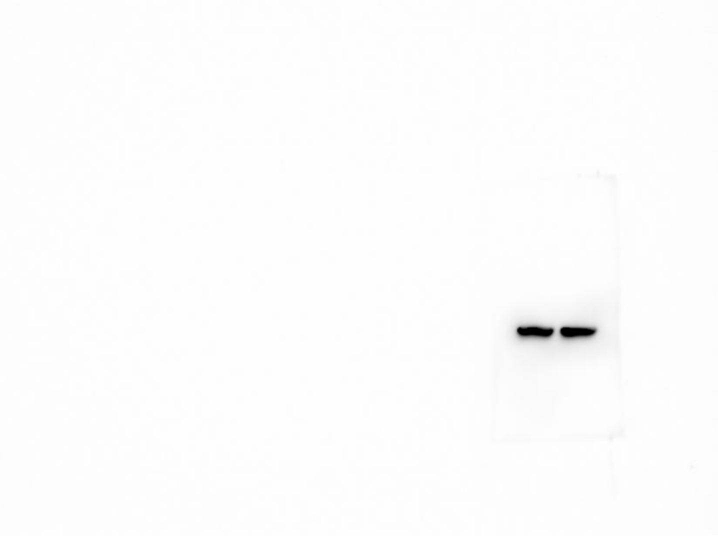

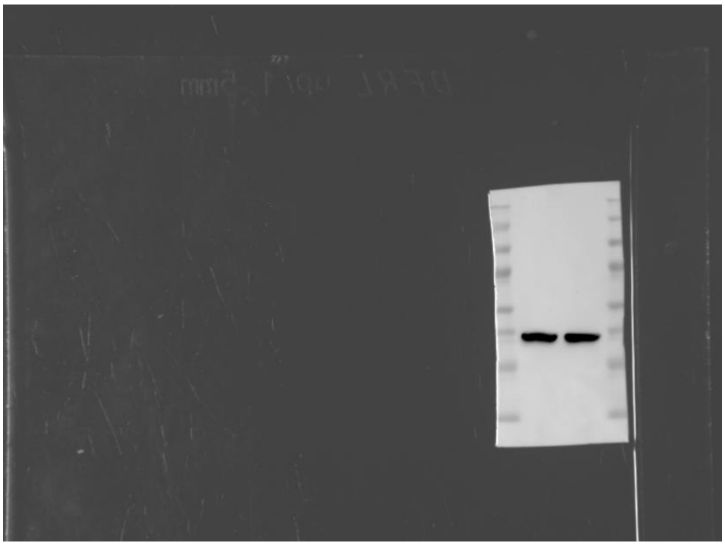

Fig 3H HepG2 G6PD

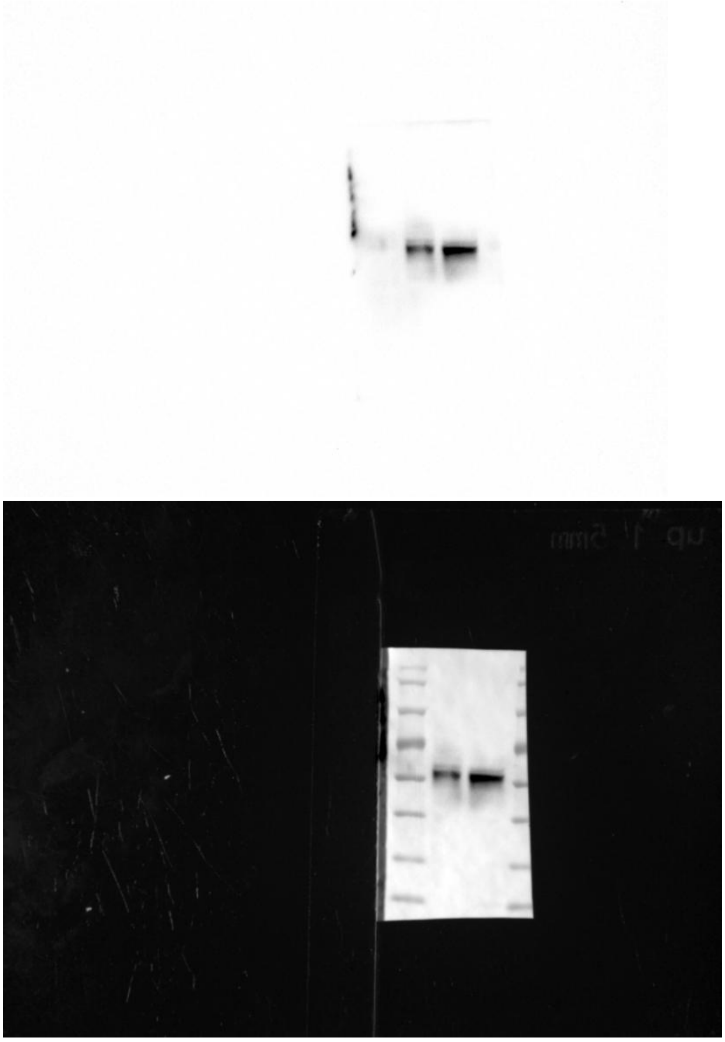

Fig 3H HepG2-AKR1B10

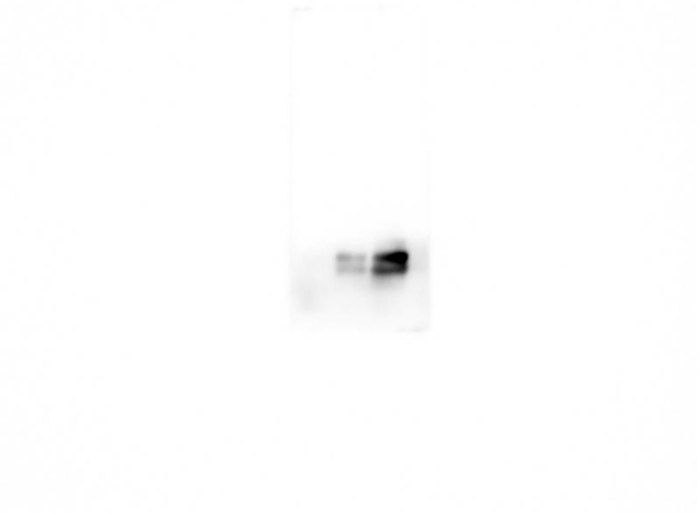

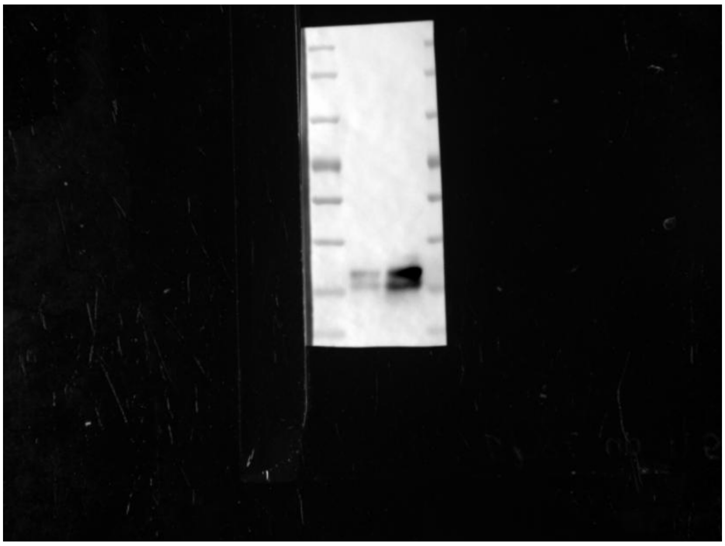

Fig 3H HepG2 Actin

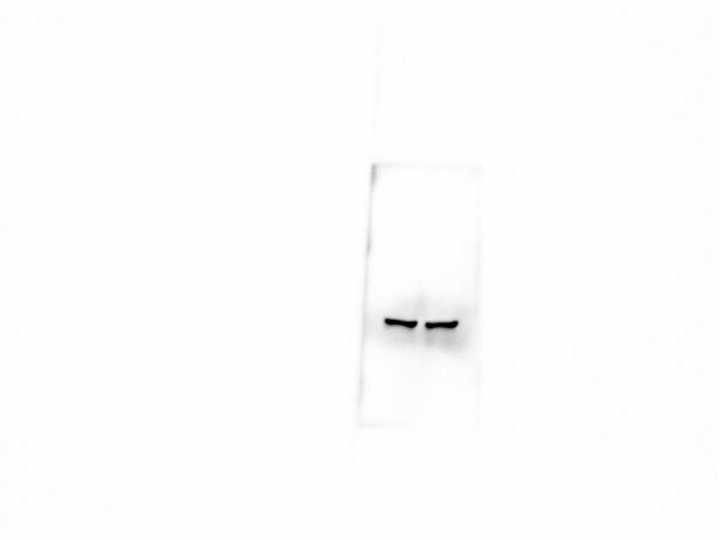

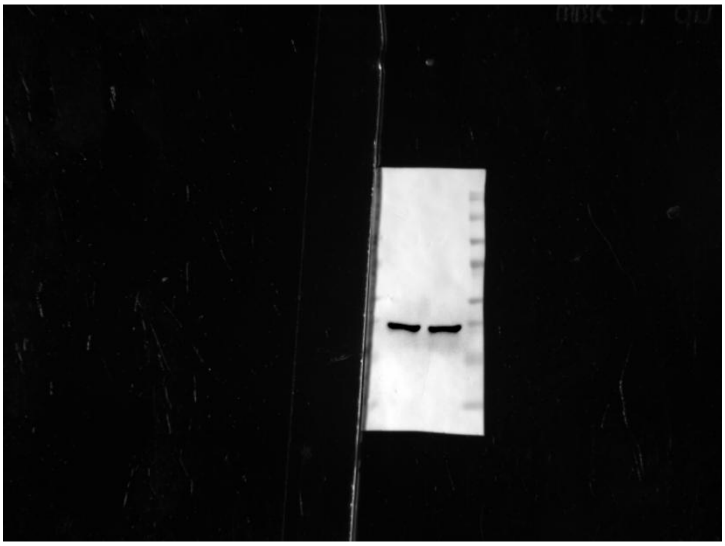

Fig 6B HCC-LM3-ITGB1

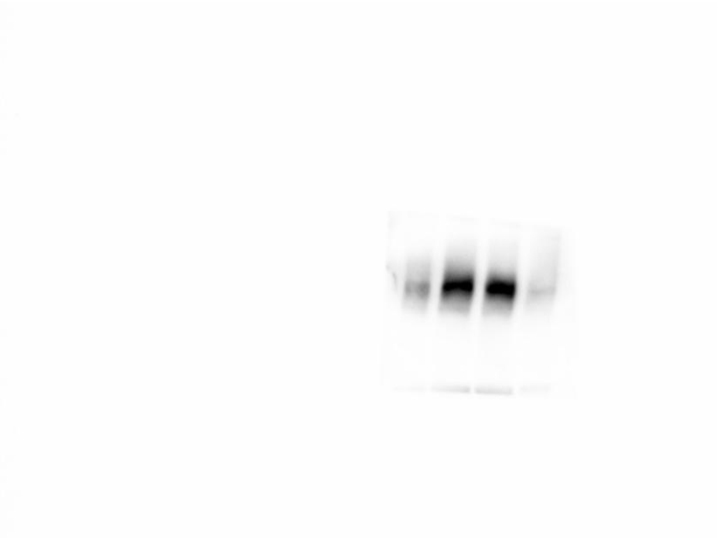

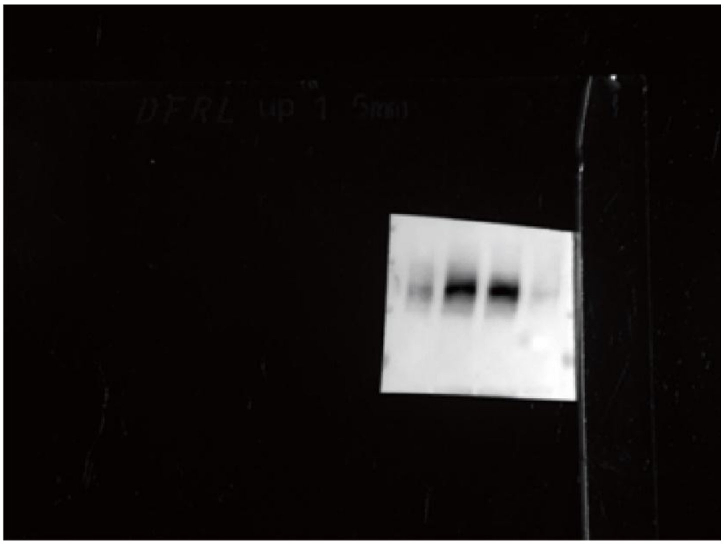

Fig 6B HCC-LM3 -PS473-AKT

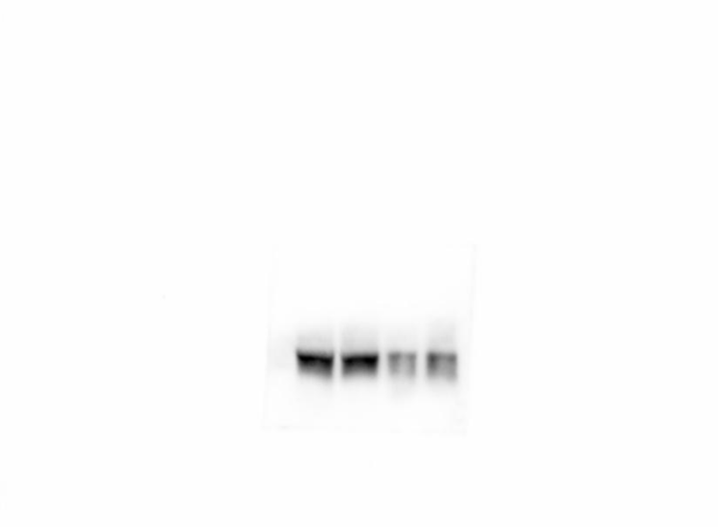

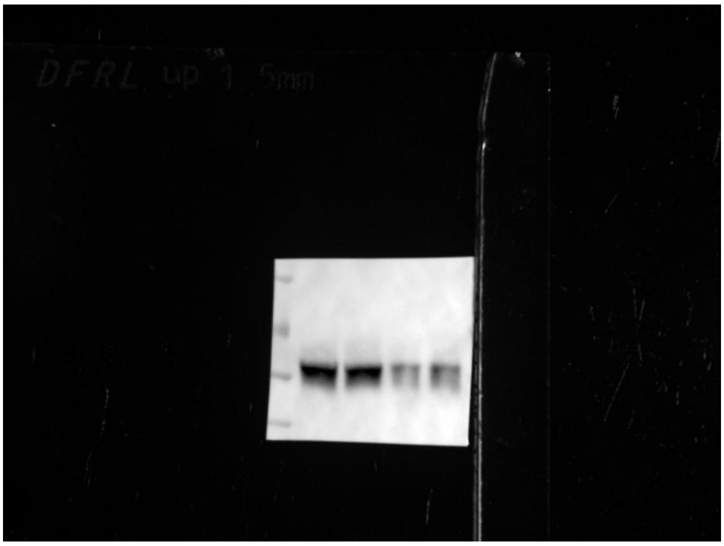

Fig 6B HCC-LM3-total-AKT

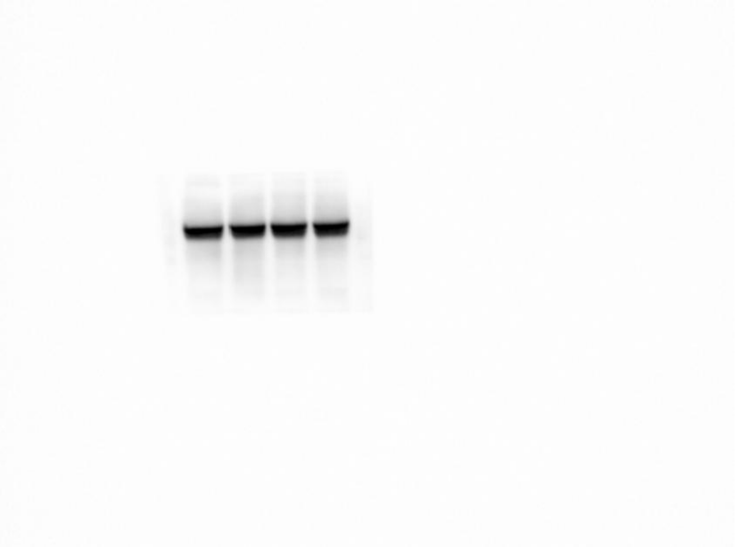

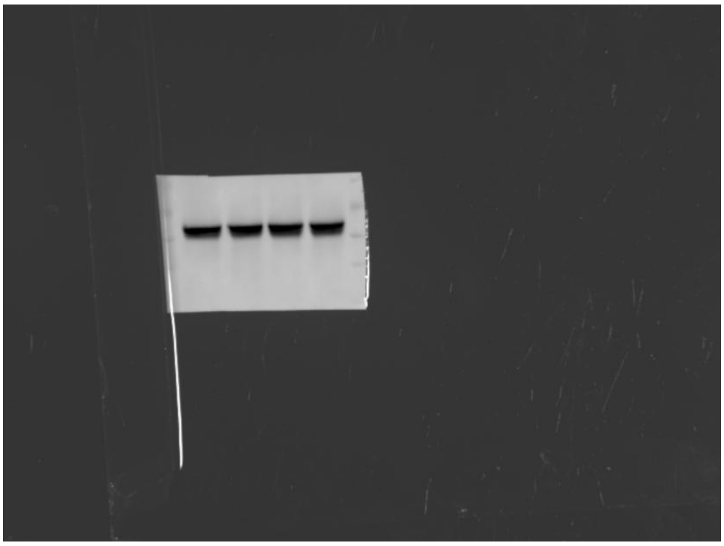

Fig 6B HCC-LM3 G6PD

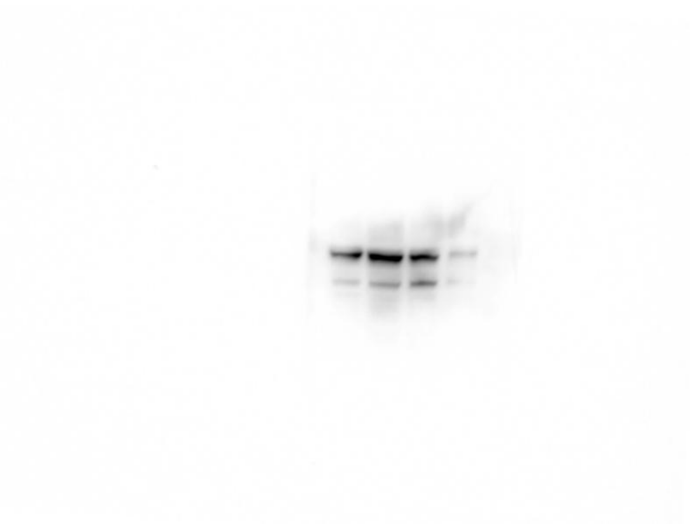

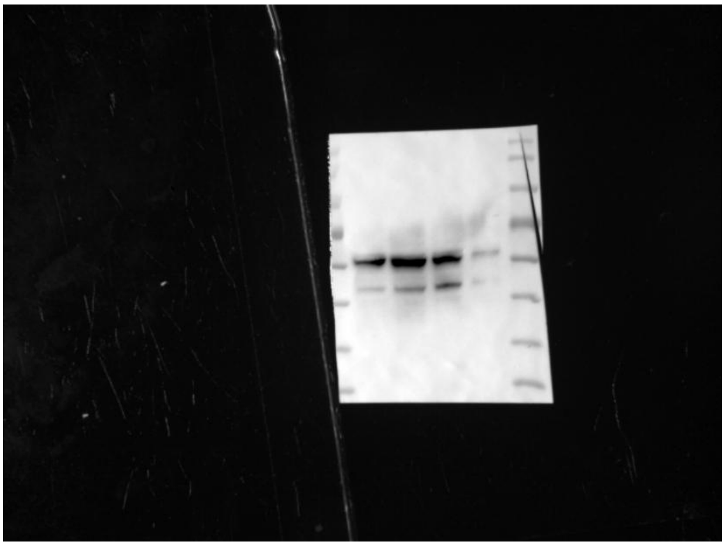

Fig 6B HCC-LM3 Actin

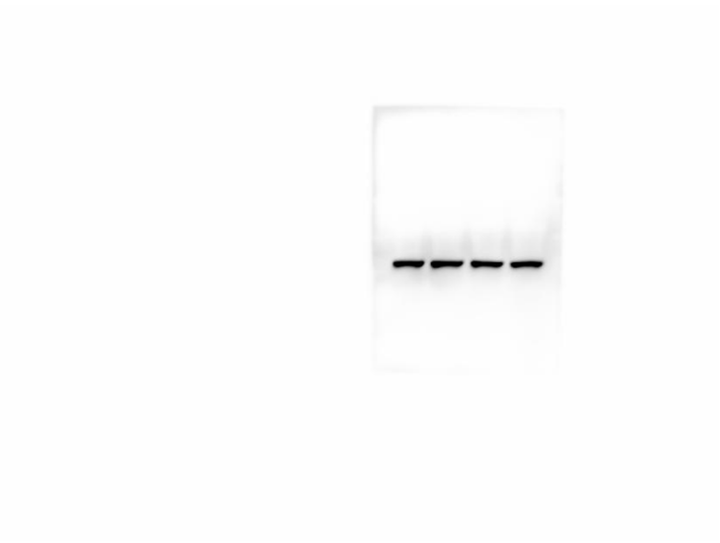

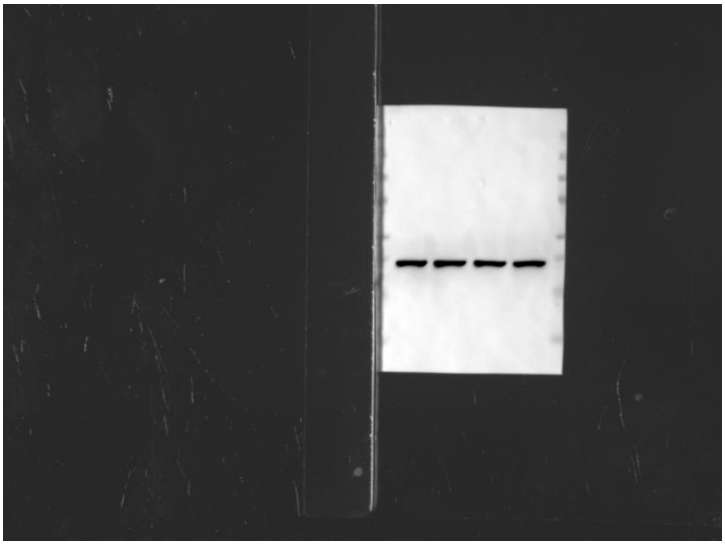

Fig 6B HepG2-ITGB1

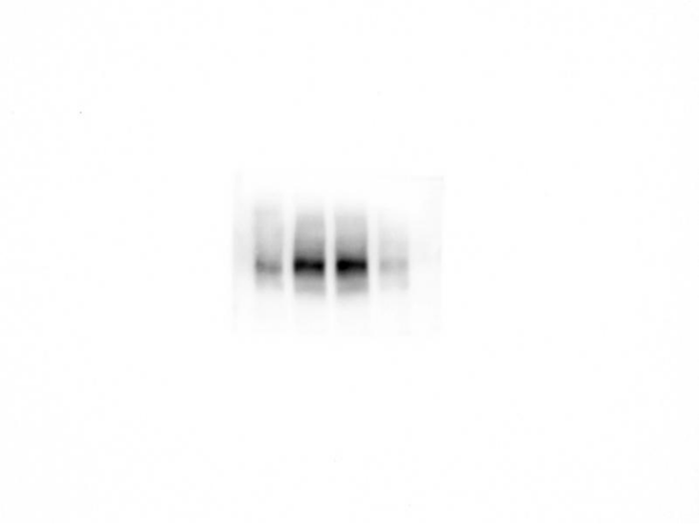

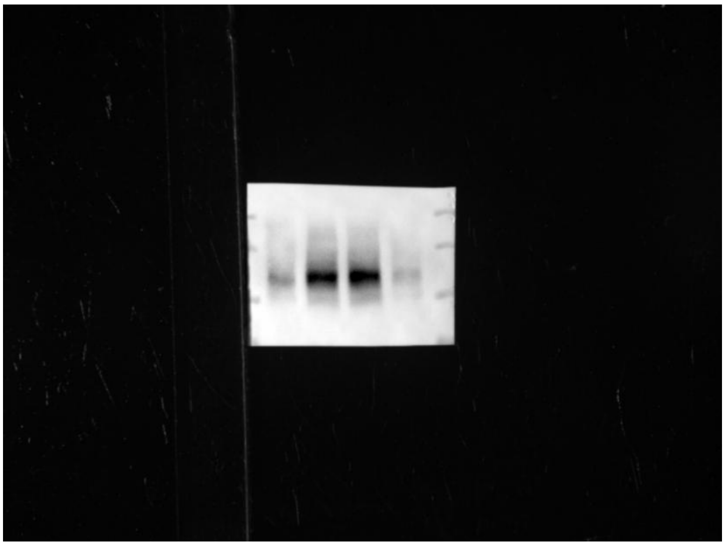

Fig 6B HepG2-PS473-AKT

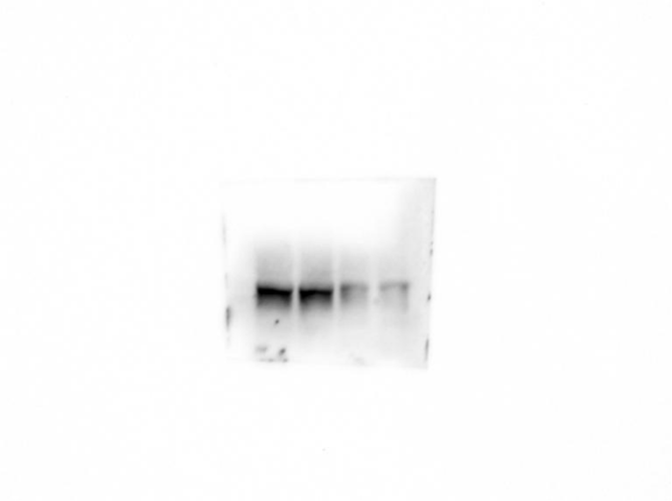

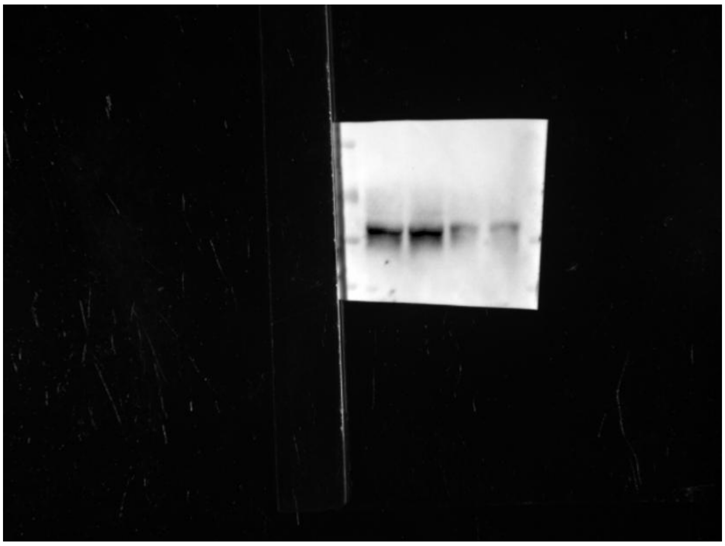

Fig 6B HepG2-total AKT

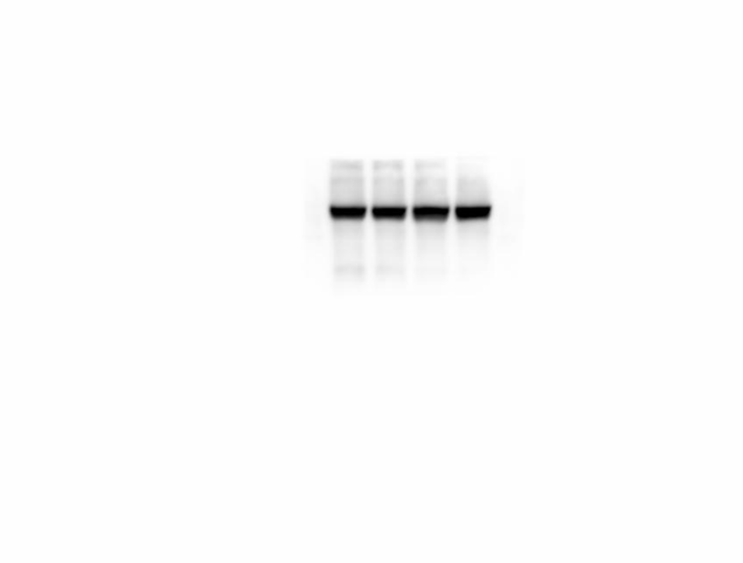

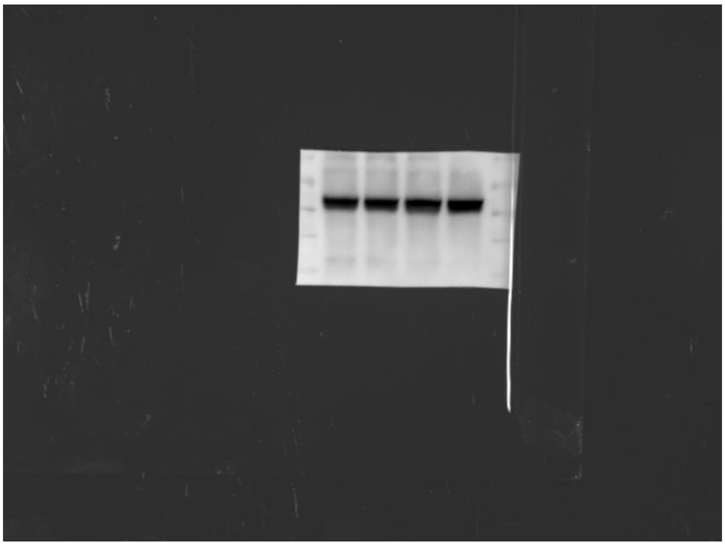

Fig 6B HepG2 G6PD

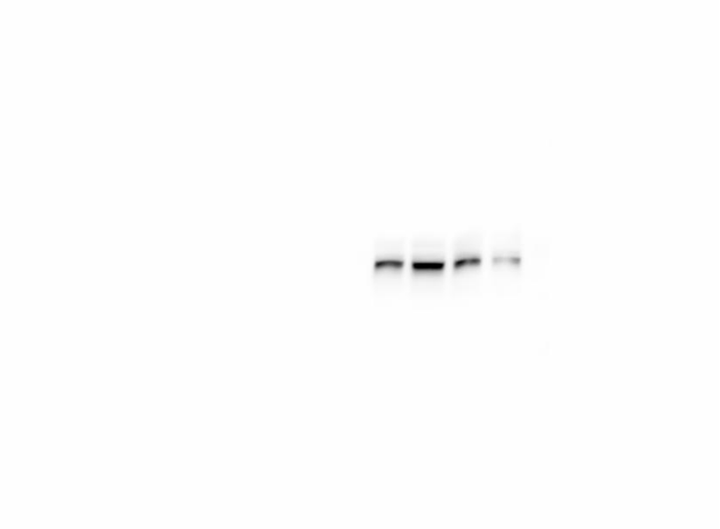

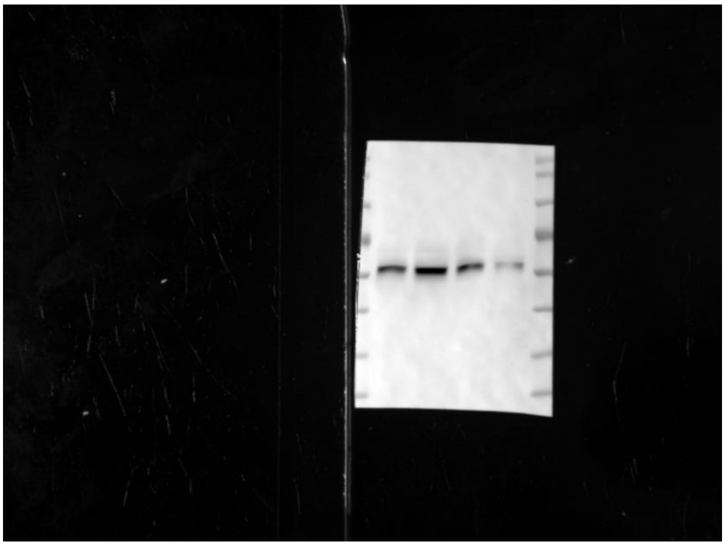

Fig 6B HepG2-Actin

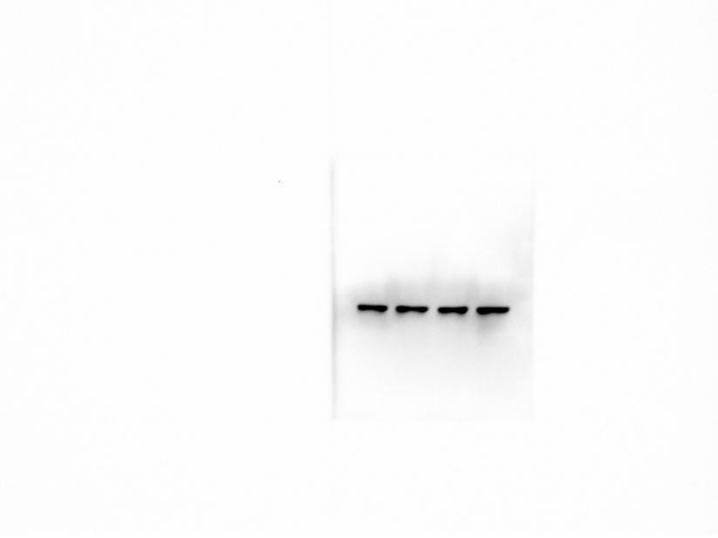

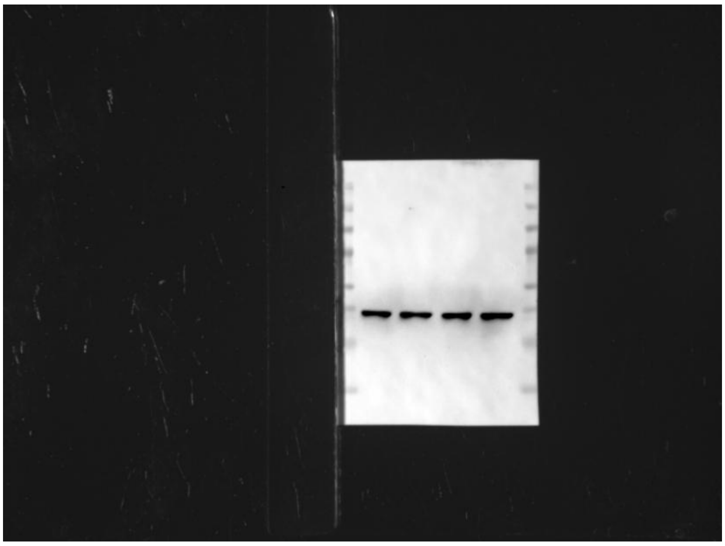

Fig S3B- HCC-LM3 G6PD

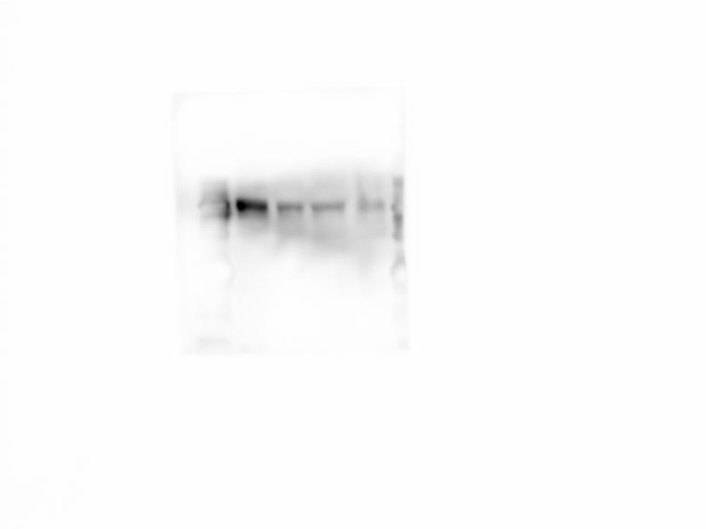

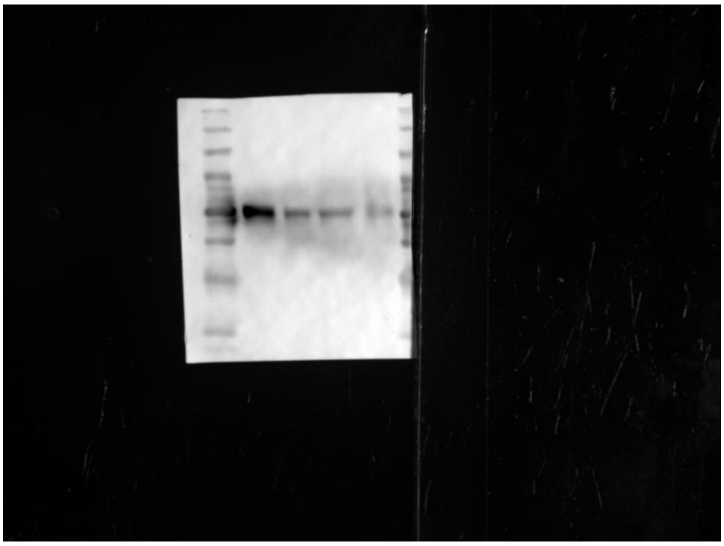

Fig S3B HCC-LM3 Actin

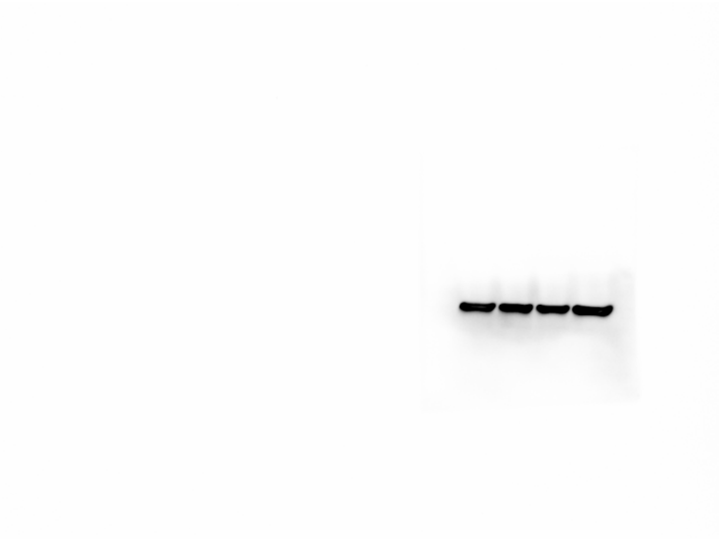

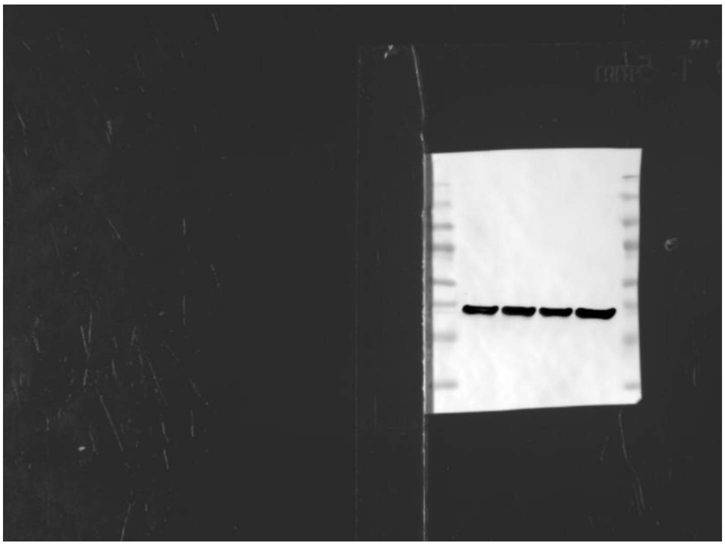

Fig S3B HepG2-G6PD

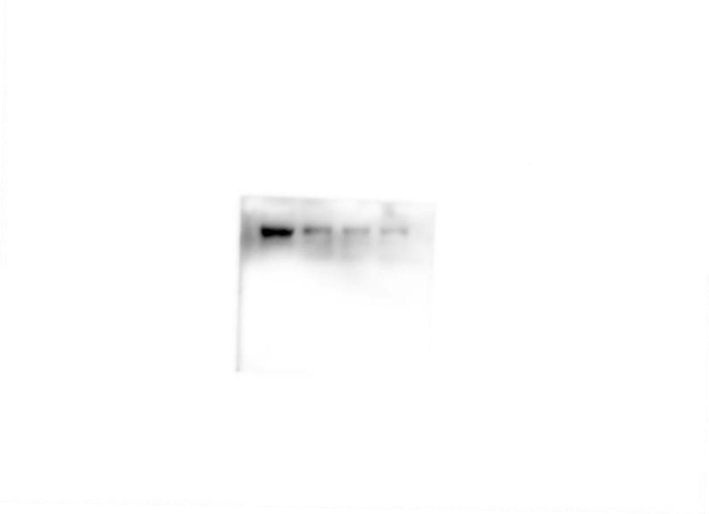

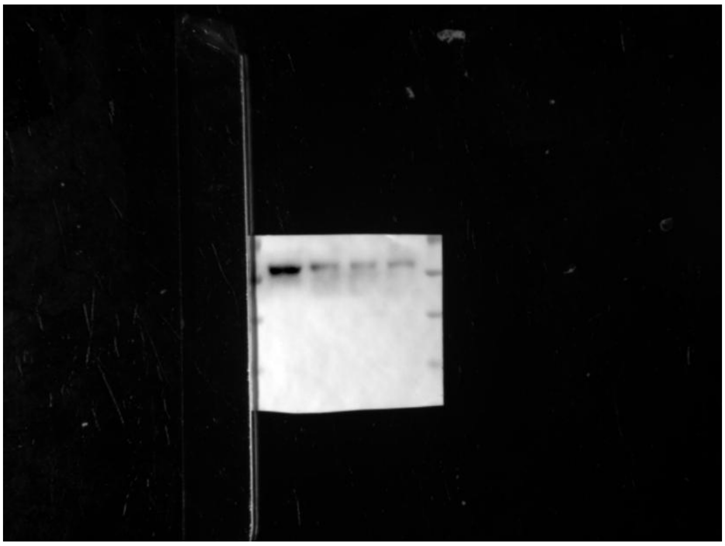

Fig S3B HepG2-ACTIN

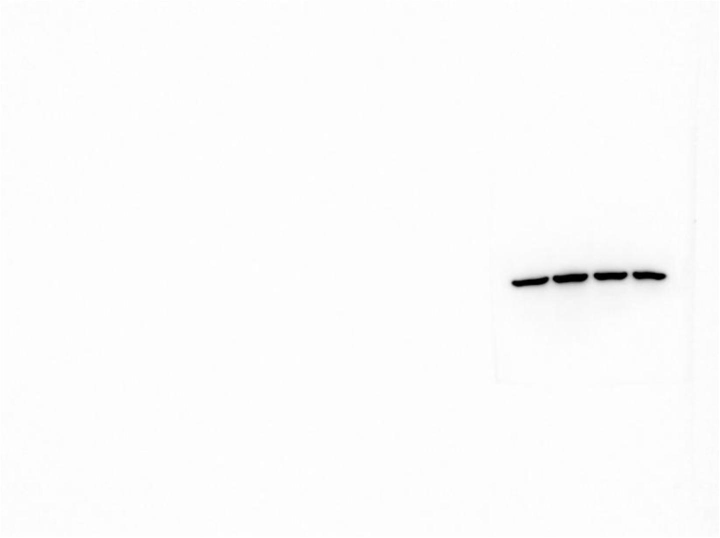

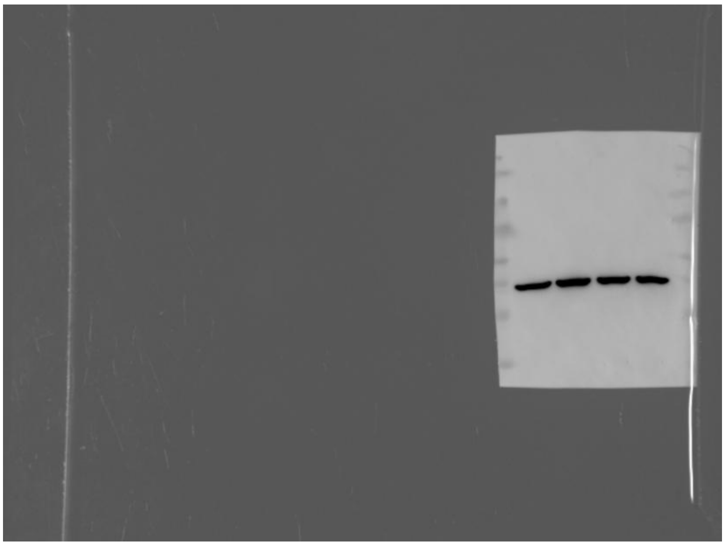

Fig S3D HCC-LM3 G6PD

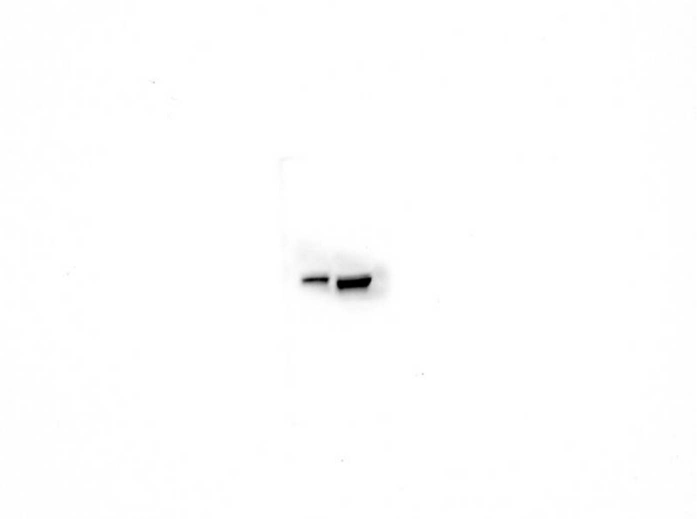

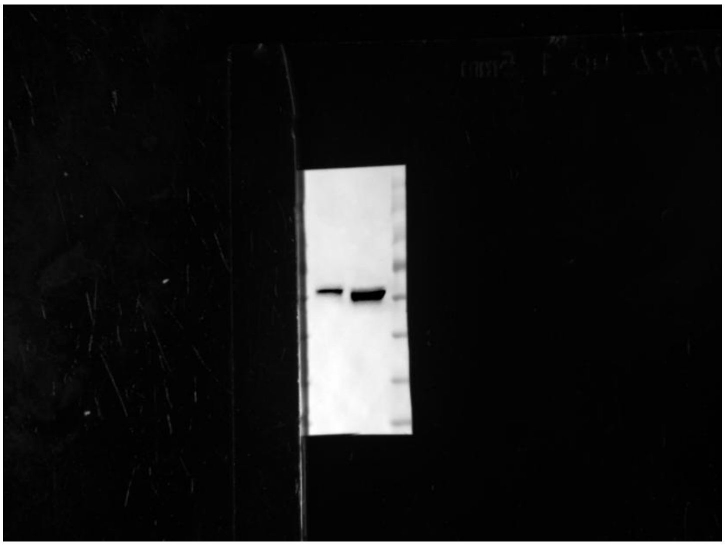

Fig S3D HCC-LM3 Actin

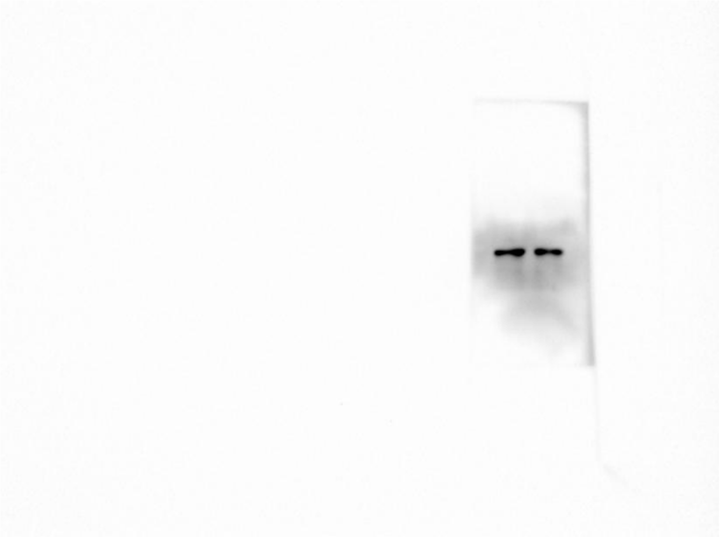

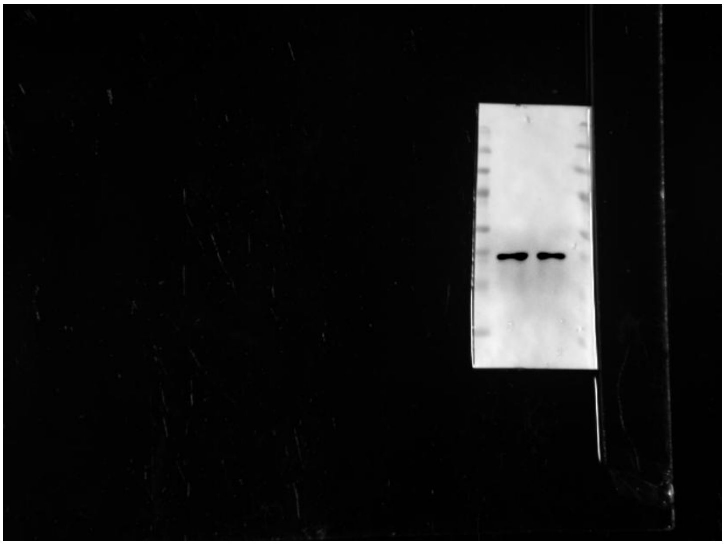

Fig S3D HepG2 G6PD

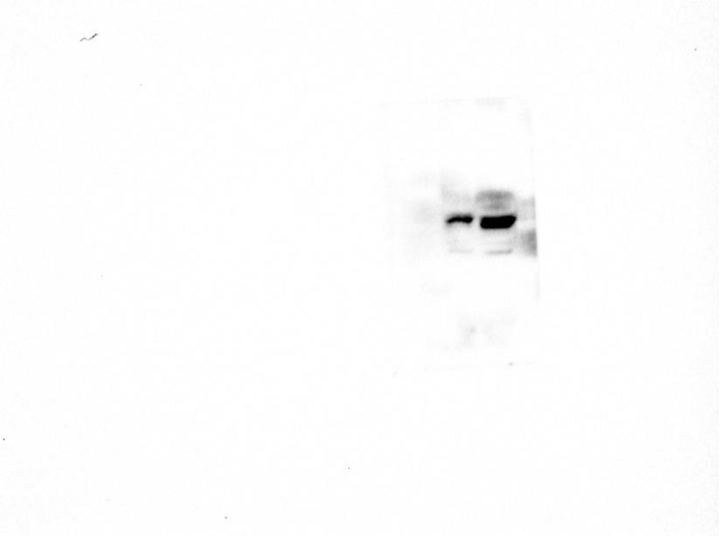

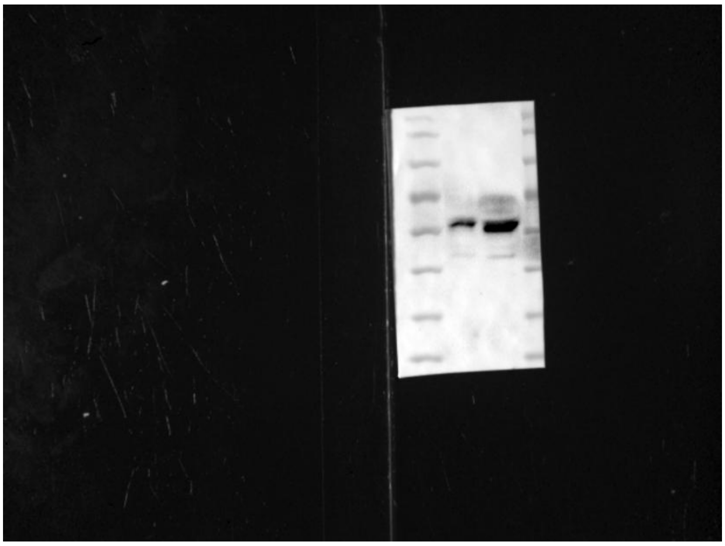

Fig S3D HepG2-Actin

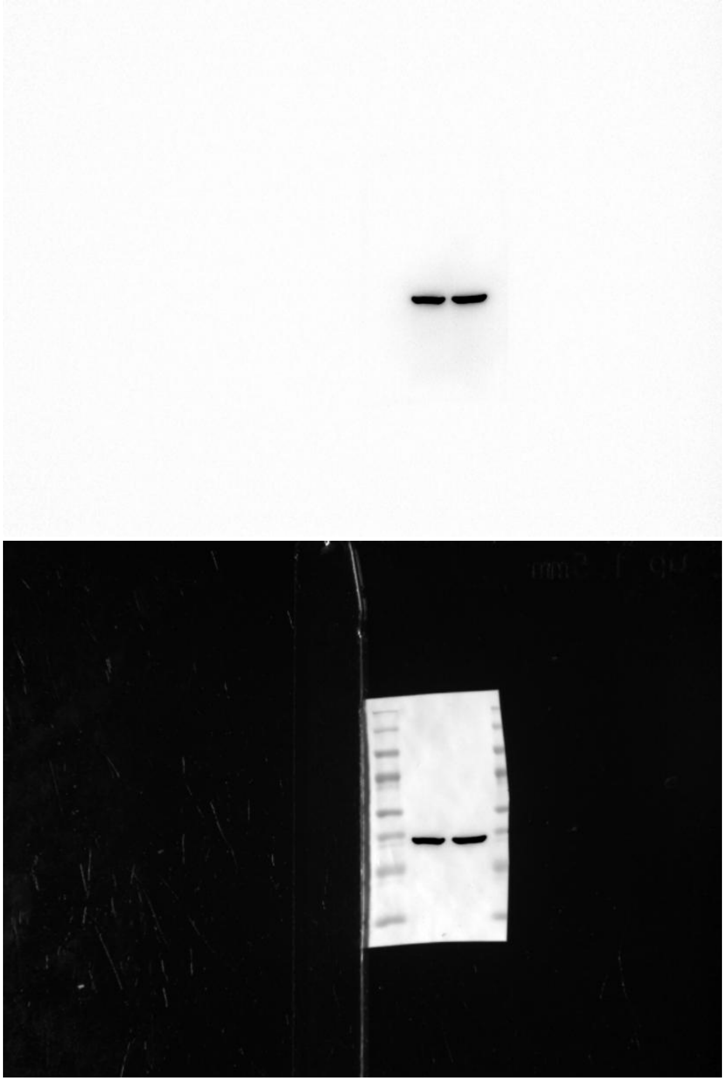

Supplement: Supplementary file 2 — WB Raw data [file 41419_2025_7842_MOESM2_ESM.pdf]
